# From Wild-Growing Plants to Promising Candidates for Hemp Variety Development: Morphological and Phytochemical Characterization of Macedonian *Cannabis sativa* L.

**DOI:** 10.3390/molecules31203723

**Published:** 2026-10-16

**Authors:** Veronika Stoilkovska Gjorgievska, Ivana Cvetkovikj Karanfilova, Nikola Geskovski, Ana Trajkovska Matevska, Iskra Davkova Stojanovski, Filip Todorov, Marija Karapandzova, Gjoshe Stefkov

**Affiliations:** 1 Institute of Pharmacognosy, Faculty of Pharmacy, Ss. Cyril and Methodius University in Skopje, Majka Tereza 47, 1000 Skopje, North Macedonia; ivanacvetkovikj@ff.ukim.edu.mk (I.C.K.); ana.trajkovska@ff.ukim.edu.mk (A.T.M.); iskradavkova@ff.ukim.edu.mk (I.D.S.); fito@ff.ukim.edu.mk (F.T.); marija_karapandzova@ff.ukim.edu.mk (M.K.); 2 Institute of Pharmaceutical Technology, Faculty of Pharmacy, Ss. Cyril and Methodius University in Skopje, Majka Tereza 47, 1000 Skopje, North Macedonia; ngeskovski@ff.ukim.edu.mk

**Keywords:** UPOV descriptors, chemotype, cannabidiol (CBD), phytocannabinoids, agronomic traits, PCA, PLS-DA

## Abstract

Background: Wild-growing *Cannabis sativa* L. represents an underexplored source of hemp-type plant material, but its value for future selection depends on standardized morphological and phytochemical characterization. This study evaluated cultivated Macedonian *Cannabis sativa* plants of wild-origin (CsW) in comparison with selected commercial cultivars and literature-reported European hemp varieties, based on the assessment of phenotypic differentiation and cannabinoid diversity. Methods: Seeds from five wild-growing plant groups from two N. Macedonian regions, Kočani (W1, W2, W6) and Strumica (W3 and W4), were planted outdoors together with four commercial cultivars with differing cannabinoid composition. Morphological traits were assessed using selected UPOV descriptors and multivariate analysis. Cannabinoids were determined by HPLC-DAD. Results: UPOV-based PCA clearly differentiated CsW from the commercial cultivars (R^2^X = 0.92, Q^2^ = 0.86), while the expanded comparative UPOV analysis indicated phenotypic affinity with registered hemp-type material, particularly Kompolti, supporting its potential relevance as a phenotypic reference in future DUS-oriented evaluation. Among the cultivated wild-origin groups, W1 from the Kočani region showed the highest proportion of female plants (10:1), representing a favorable agronomic characteristic for flower-oriented production. All CsW were CBD-predominant chemotype III class, with total CBD of 5.14–7.61%, while total Δ9-THC did not exceed 0.20%. This CBD/THC balance positioned the CsW within the higher-CBD range reported for selected European hemp cultivars, while maintaining a favorable low-THC profile. Conclusions: CsW represent morphologically distinct, CBD-rich and low-THC plant material displaying characteristics consistent with a hemp-type profile. The combined UPOV and cannabinoid data support its relevance as a promising resource for further agronomic evaluation, phytochemical selection and future registration-oriented hemp research.

## 1. Introduction

*Cannabis sativa* L. is a multipurpose plant species with increasing relevance in industrial, medicinal and pharmaceutical sectors [1,2]. Its morphological and phytochemical diversity is shaped by genetic background, environmental conditions, geographical origin and cultivation practices [3,4,5]. Consequently, the use of well-defined plant material is essential for obtaining reproducible data and for comparing results across studies. In Europe, research on low-THC *Cannabis sativa* L. has largely focused on officially listed industrial hemp cultivars [6], including Futura 75, Fedora 17, Felina 32, Finola, Santhica 27, USO 31, Kompolti, Carmagnola, Bialobrzeskie and KC Dóra [7,8,9,10,11,12]. These cultivars are commonly used in agronomic, phytochemical and breeding studies because they provide a known origin, certified seed material, documented agronomic or chemical traits, improved uniformity and predictable performance under field or controlled cultivation conditions [7,8,9,10,11,12].

In contrast, studies on medicinal and commercial cannabis often include market-derived chemovars, hybrids, or breeder-reported strain names that are not necessarily linked to formal variety registration or harmonized descriptors [13,14]. The increasing number of officially registered varieties, together with the expansion of commercial strain databases, reflects growing breeding, regulatory, and market interest in cannabis. However, official variety registers and commercial databases differ in purpose and standardization: the former support legal variety registration, protection, and standardized testing, whereas the latter primarily document commercially available hybrids, breeder-reported names, genetic lineages, and, in some cases, chemovar characteristics [6]. Thus, commercial databases provide useful information on market diversity and breeding trends but are not equivalent to officially registered varieties.

The use of officially registered varieties has promoted greater standardization in cannabis research, facilitating comparisons of agronomic performance, cannabinoid composition, and environmental adaptation. This distinction is particularly relevant when characterizing less formalized material, including wild-growing cannabis.

Against this background, UPOV-based descriptors provide a standardized framework for characterizing both registered varieties and non-registered plants. UPOV guidelines define harmonized descriptors covering morphological traits, sex expression, flowering time, plant architecture, and cannabinoid-related characteristics, supporting reliable identification and comparison of cannabis material. Although the present study does not aim at variety registration, these descriptors were used to provide a structured basis for characterizing Macedonian wild-growing cannabis, comparing it with cultivated reference material and registered cannabis varieties reported in the literature.

Despite the long history of hemp cultivation in Europe, the historical distribution and cultivation of cannabis in parts of the Balkan region remain incompletely documented, with limited information available on the locations of wild-growing plants and the accessibility of collection sites [15]. More broadly, historical prohibition of Cannabis constrained systematic scientific research and contributed to gaps in its scientific documentation [16]. Wild-growing cannabis plants have been identified across the territory of N. Macedonia but their origin and biological status remain insufficiently clarified [17]. At present, there is insufficient evidence to classify these plants as autochthonous, domesticated or relicts of previously domesticated populations [17]. Their interpretation remains complex, since such material may originate from escaped cultivation, historical hemp cultivation or wild populations. Nevertheless, these plants may preserve traits associated with adaptation to local environmental conditions, resilience and suitability for outdoor cultivation. Hence, systematic morphological and phytochemical characterization is necessary to assess their potential contribution to future selection, breeding and cultivar development [14,15].

To date, Macedonian wild-origin cannabis has not been comprehensively characterized using an integrated UPOV-based morphological and phytochemical approach under common outdoor cultivation conditions. This may help assess its potential as a resource for hemp variety development.

Therefore, the aim of this study was to characterize cultivated Macedonian *Cannabis sativa* plants of wild-origin (CsW) through UPOV-based morphological assessment and phytochemical profiling, in comparison with selected commercial cultivars and literature-reported data for registered hemp varieties, in order to evaluate their potential as material for further hemp selection and development.

## 2. Results and Discussion

### 2.1. UPOV-Based Morphological Characterization

Morphological characterization was performed according to selected UPOV descriptors to compare the CsW with commercial cultivars, with both groups grown from seed and cultivated outdoors under the same conditions. The evaluated descriptors included cotyledon traits, leaf morphology, stem characteristics, plant height, proportion of female plants and flower yield. Detailed seed morphometric characteristics and ATR-IR-based differentiation of the investigated seed material have been reported previously [17] and are therefore not presented again here. However, the relevant UPOV seed descriptors were retained as complementary variables in the integrated multivariate analysis together with the vegetative and agronomic traits. Summary UPOV qualitative and quantitative descriptor data are provided in Table 1 whereas individual plant UPOV measurements for leaf traits, plant height and flower yield are given in Supplementary Tables S1 and S2.

Among the evaluated quantitative traits, plant height varied across the investigated groups, ranging from 122.75 cm in NYC Diesel to 235.25 cm in W3. CsW generally showed greater mean plant height than the commercial cultivars under the same outdoor cultivation conditions. Leaf morphology also varied among the investigated groups, with the largest mean central leaflet dimensions recorded in Charlotte’s Angel, reaching 57.87 mm in length and 4.22 mm in width. Among the CsW, W2 and W4 had greater mean central leaflet lengths compared with W1 and W3, whereas W3 showed the lowest mean central leaflet width. Overall, the descriptive data showed variability in plant stature and leaf morphology among the investigated groups under common outdoor cultivation conditions.

Mean flower yield varied among the investigated groups, ranging from 56.75 g (GWS) to 92.67 g (CA). W2 and W1 had mean flower yields of 89.46 g and 84.43 g, respectively. Thus, W1 and W2 were among the groups with the highest mean flower biomass recorded across the investigated plant groups.

The distribution of female and male plants varied among the cultivated Macedonian *Cannabis sativa* groups of wild-origin, with W1 showing the strongest female predominance (10:1 female-to-male ratio), whereas all commercial cultivars consisted exclusively of female plants. The pronounced female-biased ratio observed in W1 may represent a potentially favorable characteristic for flower-oriented cultivation, where a high proportion of female plants is desirable. Nevertheless, as this ratio was observed during a single cultivation experiment, its stability remains to be confirmed. Although sex determination in dioecious *C. sativa* has a strong genetic basis, sex expression may also be modulated by environmental and cultivation conditions, including photoperiod and temperature [18], nutrient availability [19], and environmental stress [20]. Thus, the observed female predominance in W1 is a promising feature that warrants further evaluation across additional growing seasons and environmental conditions. An unsupervised PCA model was constructed using the morphological dataset obtained from the CsW and commercial cultivars, to determine whether the recorded UPOV descriptors provide sufficient discriminatory information beyond descriptive morphology. The model showed strong explanatory and predictive performance, with R^2^X = 0.92 and Q^2^ = 0.86. Five principal components explained 92.33% of the total variability, with PC1, PC2, PC3, PC4 and PC5 accounting for 35.52%, 22.77%, 18.66%, 9.29% and 6.09% of the variance, respectively.

**Figure 1:**
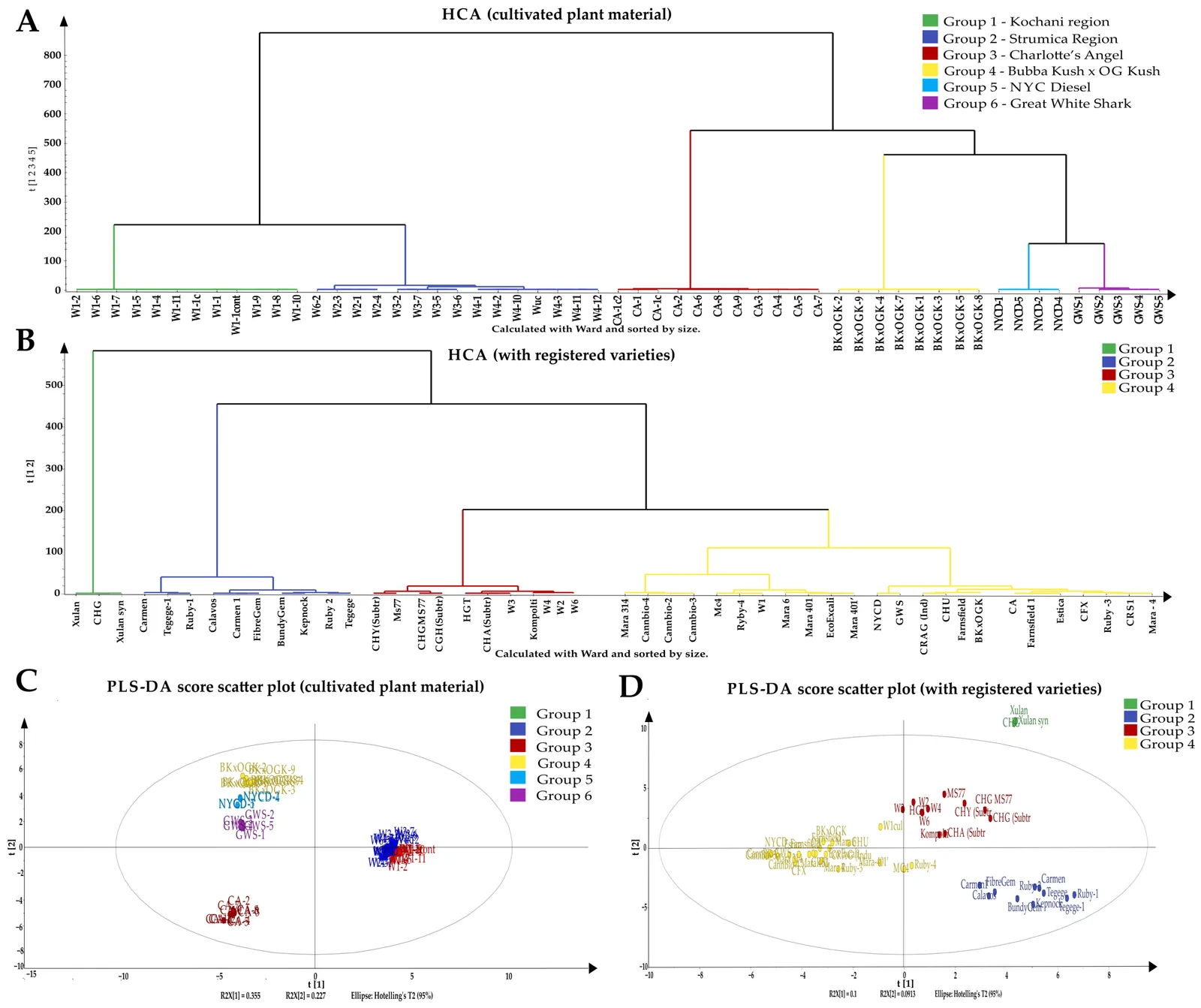
Hierarchical cluster analysis (HCA) (**A**) and PLS-DA score scatter plot (**C**) based on UPOV morphological descriptors of CsW and commercial cultivars; HCA (**B**) and PLS-DA score scatter plot (**D**) based on the expanded UPOV dataset, including CsW, commercial cultivars, and literature-reported registered Australian cannabis varieties. Abbreviations: HCA—hierarchical cluster analysis; PLS-DA—partial least square discriminatory analysis; W1–W6—cultivated Macedonian *Cannabis sativa* plant groups of wild-origin (CsW); CA—Charlotte’s Angel; BK × OGK—Bubba Kush × OG Kush; GWS—Great White Shark; NYC Diesel—New York City Diesel.

The score scatter plot revealed that the investigated material did not distribute randomly, but followed a clear pattern according to morphological similarity. The first principal component was mainly responsible for the separation between plants of wild-origin and commercial cultivars, indicating that their distinction was not driven by a single trait, but by the combined contribution of several UPOV descriptors. These included plant height, stem characteristics, leaf morphology, seed traits and flower-yield-related parameters. Therefore, the PCA model confirmed that the CsW possess a distinct morphological profile when compared with the commercial cultivars grown under the same outdoor conditions. The PCA score plot also indicated variation within the CsW, with partial grouping according to their original collection region, particularly between the Kočani- and Strumica-derived groups. This suggests that UPOV descriptors captured region-associated phenotypic variation within CsW. Ward’s hierarchical cluster analysis was subsequently performed using the PCA model scores (Figure 1A). The dendrogram separated the investigated material into six main clusters, supporting the distribution observed in the PCA score plot. The clustering pattern confirmed the separation between the plants of wild-origin and commercial cultivars and further indicated that the wild-growing material was not morphologically homogeneous.

Based on the HCA-derived groups, a supervised PLS-DA model was developed to evaluate the discriminatory potential of the UPOV descriptor dataset. The model comprised six latent variables and showed R^2^X = 0.94, R^2^Y = 0.99 and Q^2^ = 0.99. Internal model validation was performed using permutation testing and CV-ANOVA. The 50-permutation test (Figure S1) yielded R^2^ and Q^2^ regression-line intercepts of 0.0491 and −0.509, respectively, while CV-ANOVA (Table S3) confirmed the statistical significance of the cross-validated model (F = 64.59, *p* < 0.001). The high R^2^Y and Q^2^ values indicate that the morphological descriptors were sufficient not only to describe the investigated material, but also to classify the predefined groups with high predictive ability. The PLS-DA score scatter plots (Figure 1C) further confirmed the relative position of the CsW and commercial plants, while the loading/weighting plots identified the descriptors contributing most strongly to group separation (Table 2).

A second multivariate analysis was performed by combining our experimental UPOV dataset with publicly available literature descriptor data for registered or previously described cannabis varieties. This expanded analysis was considered separately from the field-based morphological comparison, because it included external descriptor data and therefore served as a comparative phenotypic framework rather than a direct cultivation experiment.

The inclusion of registered varieties enabled evaluation of the plants of wild-origin in relation to established hemp-type material. In this expanded UPOV dataset, HCA separated the material into four main clusters (Figure 1B), followed by a PLS-DA model constructed from the selected HCA-derived groups (Figure 1D). The PLS-DA model was based on three principal components and showed satisfactory discriminatory ability, with R^2^X = 0.25, R^2^Y = 0.88 and Q^2^ = 0.67. Although the explained X-variance was lower than in the experimental UPOV model, the R^2^Y and cross-validated Q^2^ values indicated that the descriptor set retained discriminatory information within the expanded and more heterogeneous dataset. Internal validation by a 50-permutation test (Figure S2) yielded R^2^ and Q^2^ intercepts of 0.409 and −0.291, respectively, while CV-ANOVA (Table S3) demonstrated statistical significance of the cross-validated model (F = 9.16, *p* < 0.001). The most relevant observation from the comparative analysis was the morphological proximity of the CsW to Kompolti, a Hungarian hemp variety. In the HCA (Figure 1B), W2, W3, W4, and W6 clustered within the same major group as Kompolti and CHA, whereas W1 was positioned in a separate cluster together with MC4 and Ruby. Within this major cluster, W2, W4, and W6 showed particularly close clustering with Kompolti. The association of most Macedonian plants of wild-origin with Kompolti is particularly noteworthy considering the historical and regulatory importance of this variety in European hemp cultivation and its long history of cultivation and registration, with its first variety application dating back to 1952 [15,16].

Its importance is also reflected in the subsequent registration of related varieties, including Kompolti hybrid TC in Hungary in 1979 and Kompolti sárgaszárú in 1994, as well as later registration and application records involving Kompolti in several European countries [15]. Importantly for the present morphological comparison, Kompolti was included as an example variety for several states of expression in the UPOV guideline for *Cannabis sativa* L., including number of leaflets, length and width of the central leaflet, time of male flowering, main stem color and thickness, and seed marbling [21]. Furthermore, Kompolti has been used as a comparator in the examination of registered cannabis varieties within the Australian Plant Breeder’s Rights system [22]. Its position among the closest registered varieties in the present multivariate analysis, together with its established use in UPOV-based characterization and variety examination, supports its relevance as a phenotypic reference for interpreting the CsW. Interestingly, some morphological characteristics reported for Kompolti when used as a comparator in the Australian variety examination [22] differed from the states of expression assigned to Kompolti in the UPOV guideline [21], particularly for leaflet number, central leaflet dimensions, time of male flowering, and main stem color. Despite their overall proximity to Kompolti in the multivariate analysis, cultivated *Cannabis sativa* plants of wild origin showed differences in several individual UPOV descriptors. In particular, they were characterized by few leaflets compared with the medium number of leaflets reported for Kompolti, long plants compared with the medium natural height of Kompolti, and a yellow main stem compared with the green main stem of Kompolti.

Nevertheless, the consistent positioning of most CsW within the same broader phenotypic space as Kompolti indicates substantial phenotypic affinity with established hemp-type material. However, the groups did not completely overlap with Kompolti or with the other registered varieties included in the models; therefore, this association should be interpreted as morphological similarity rather than varietal identity. Moreover, because the external UPOV descriptor data were derived from literature sources representing different environments, years, cultivation conditions, and potentially different scoring procedures, direct comparison with the experimentally evaluated material is inherently limited. Accordingly, the expanded UPOV analysis should be regarded primarily as an exploratory phenotypic comparison. At the same time, this multivariate approach provides a useful basis for identifying phenotypically similar registered varieties that may serve as relevant comparators in subsequent DUS-oriented evaluation, with Kompolti emerging as one potential comparator based on the present exploratory analysis.

Another noteworthy characteristic was the pronounced female predominance observed in W1, with 10 female plants and one male plant (10:1 female-to-male ratio), with male and female plants shown in Supplementary Figures S4 and S5, respectively. This contrasts with the approximately balanced female-to-male distribution generally associated with dioecious hemp [21]; Kompolti, the principal phenotypic comparator identified in the present analysis, is itself a dioecious hemp variety [23]. At the same time, the comparative UPOV dataset showed variation in sex expression among registered hemp varieties, with Eco Excalibur, HGT, Mara 314, and Mara 401 characterized by medium-to-high proportions of female plants and low-to-medium proportions of male plants, whereas several other registered varieties showed medium proportions of both female and male plants [22]. Female-biased distributions have also been reported experimentally among commercial hemp cultivars; for example, CFX-1 comprised 82.9% female and 17.1% male plants [24]. Thus, although female-biased sex distributions are also represented among registered and commercial hemp cultivars, the 90.9% female proportion observed experimentally in W1 represents a pronounced female predominance and may constitute a potentially favorable characteristic for flower-oriented cultivation. Nevertheless, its stability requires confirmation across additional growing seasons and environmental conditions. Accordingly, the comparative UPOV analysis was used to establish the phenotypic position of the CsW relative to established varieties and to identify relevant phenotypic references for their further evaluation, rather than to assign them to an existing variety or define a new taxonomic entity. Overall, the experimental and comparative UPOV-based analyses indicate that the CsW are morphologically differentiated from the high-cannabinoid commercial cultivars while showing closer affinity to registered hemp-type material, including Kompolti within the exploratory comparative analysis.

### 2.2. Cannabinoid Profile

The cannabinoid profile of dried female flowers was determined by HPLC-DAD through quantification of CBDA, CBD, CBN, Δ9-THC and Δ9-THCA, while total CBD and total Δ9-THC were calculated from the corresponding acidic and neutral forms. Individual results are presented in Supplementary Tables S5 and S6. In all cultivated Macedonian *Cannabis sativa* plant groups of wild-origin, CBDA was the predominant cannabinoid, whereas Δ9-THCA and Δ9-THC were present at low levels. Consequently, all CsW were classified as chemotype III class [25]. Mean total CBD content was 5.15 ± 1.41% in W1, 5.14 ± 0.64% in W2, 5.88 ± 0.39% in W3 and 5.96 ± 1.18% in W4, while W6-2 showed 7.61% total CBD. Total Δ9-THC was below 0.2% in all CsW. Among the commercial cultivars, Charlotte’s Angel also showed a CBD-predominant profile, with higher mean total CBD content (10.73 ± 1.70%), but total Δ9-THC exceeded the low-THC hemp threshold (0.60 ± 0.53%). Bubba Kush × OG Kush [26] and NYC Diesel were Δ9-THCA/Δ9-THC-predominant and were classified as chemotype I [25], with mean total Δ9-THC contents of 9.31 ± 4.92% [26] and 9.89 ± 1.80%, respectively. Great White Shark showed an intermediate cannabinoid profile, with mean total CBD and total Δ9-THC contents of 5.24 ± 3.08% and 6.76 ± 3.28%, respectively, corresponding to chemotype II class [25]. Considerable intra-group variability was observed, particularly in W1 for total CBD and in Bubba Kush × OG Kush [26] and Great White Shark for total Δ9-THC.

**Figure 2:**
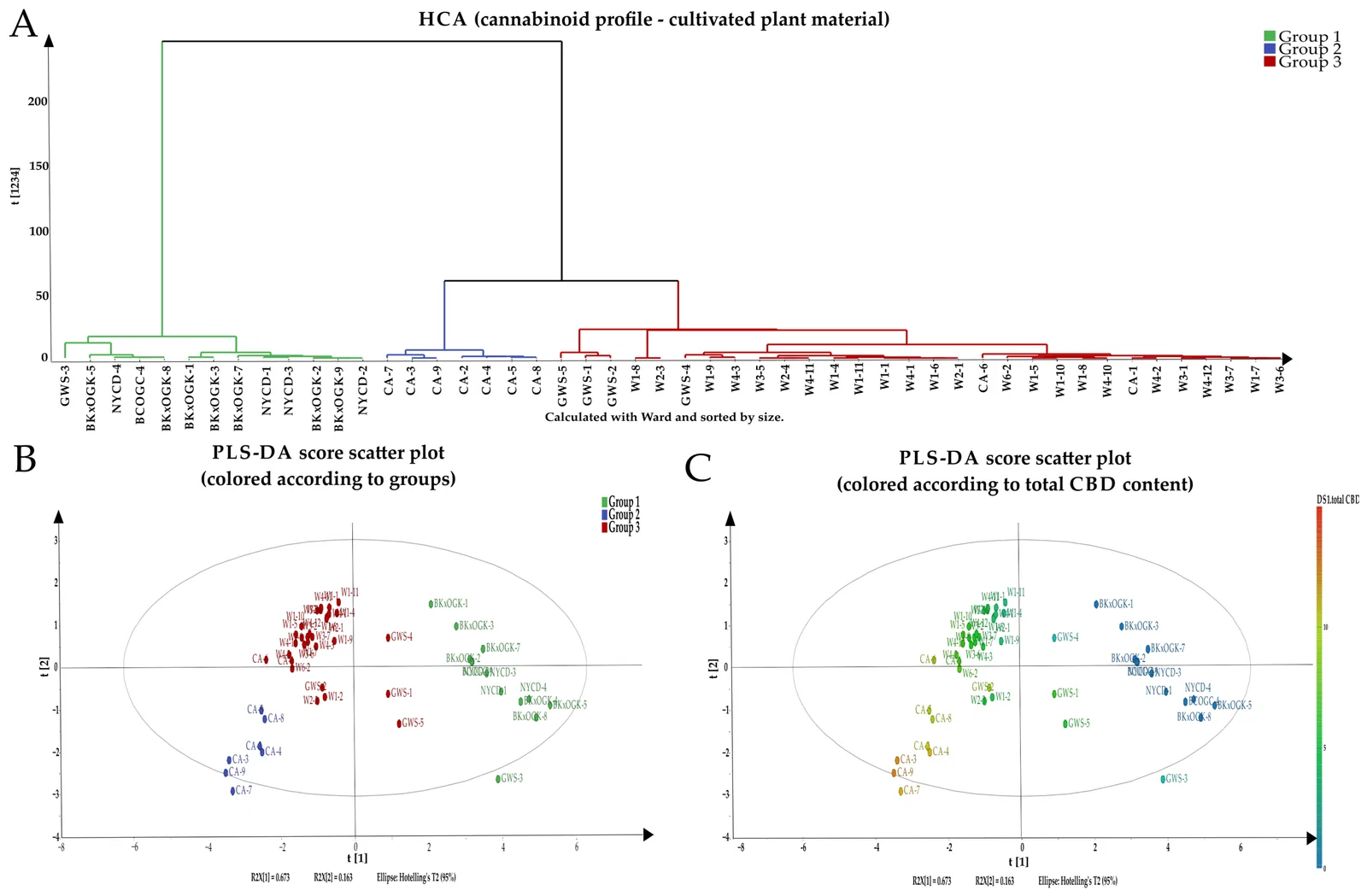
HCA (**A**); PLS-DA score scatter plots based on the cannabinoid profiles of CsW and commercial cannabis strains (**B**); with samples colored according to the total CBD content (highest content—orange; lowest content—dark blue) (**C**). Abbreviations: HCA—hierarchical cluster analysis; PLS-DA—partial least square discriminatory analysis; W1–W6—cultivated Macedonian *Cannabis sativa* plant groups of wild-origin (CsW); CA—Charlotte’s Angel; BK × OGK—Bubba Kush × OG Kush; GWS—Great White Shark; NYC Diesel—New York City Diesel.

An unsupervised PCA model was constructed using the quantified cannabinoids and calculated total cannabinoid values and showed strong explanatory and predictive performance, with R^2^X = 0.97 and Q^2^ = 0.73. Four principal components explained 97.07% of the total variability, with PC1 accounting for 67.33%, PC2 for 16.56%, PC3 for 9.45% and PC4 for 3.73%. Ward’s HCA (Figure 2A) separated the samples into three main clusters, which were subsequently used to construct a supervised PLS-DA model with three components. The PLS-DA model showed satisfactory discrimination, with R^2^X = 0.89, R^2^Y = 0.74 and Q^2^ = 0.69. Internal validation by a 50-permutation test (Figure S3) yielded R^2^ and Q^2^ intercepts of 0.003 and −0.271, respectively, while CV-ANOVA (Table S3) demonstrated statistical significance of the cross-validated model (F = 21.44, *p* < 0.001).

The score scatter plots of the PLS-DA model reveal the relative positions of the sample subsets based on the HCA clustering (Figure 2B) and total CBD contents (Figure 2C), while the loading plots (Figure S6) for each principal component identify the main variables contributing to the differentiation of the groups in the model.

The first PLS-DA component mainly separated Δ9-THC/Δ9-THCA-rich material from CBDA/CBD-rich material, whereas the second further differentiated Charlotte’s Angel from the CsW. CBN did not contribute substantially to sample differentiation. The multivariate analysis therefore confirmed that the CsW formed a separate CBDA/CBD-predominant cluster (Group 3) with total Δ9-THC not exceeding 0.20%, whereas the commercial cultivars were distributed according to their chemotype as THC-predominant, CBD- predominant or intermediate [24].

The observed Δ9-THC levels comply with the legally permitted limit in North Macedonia and with the European Union regulations applicable to *Cannabis sativa* L. plants intended for industrial and agricultural purposes [26,27].

**Figure 3:**
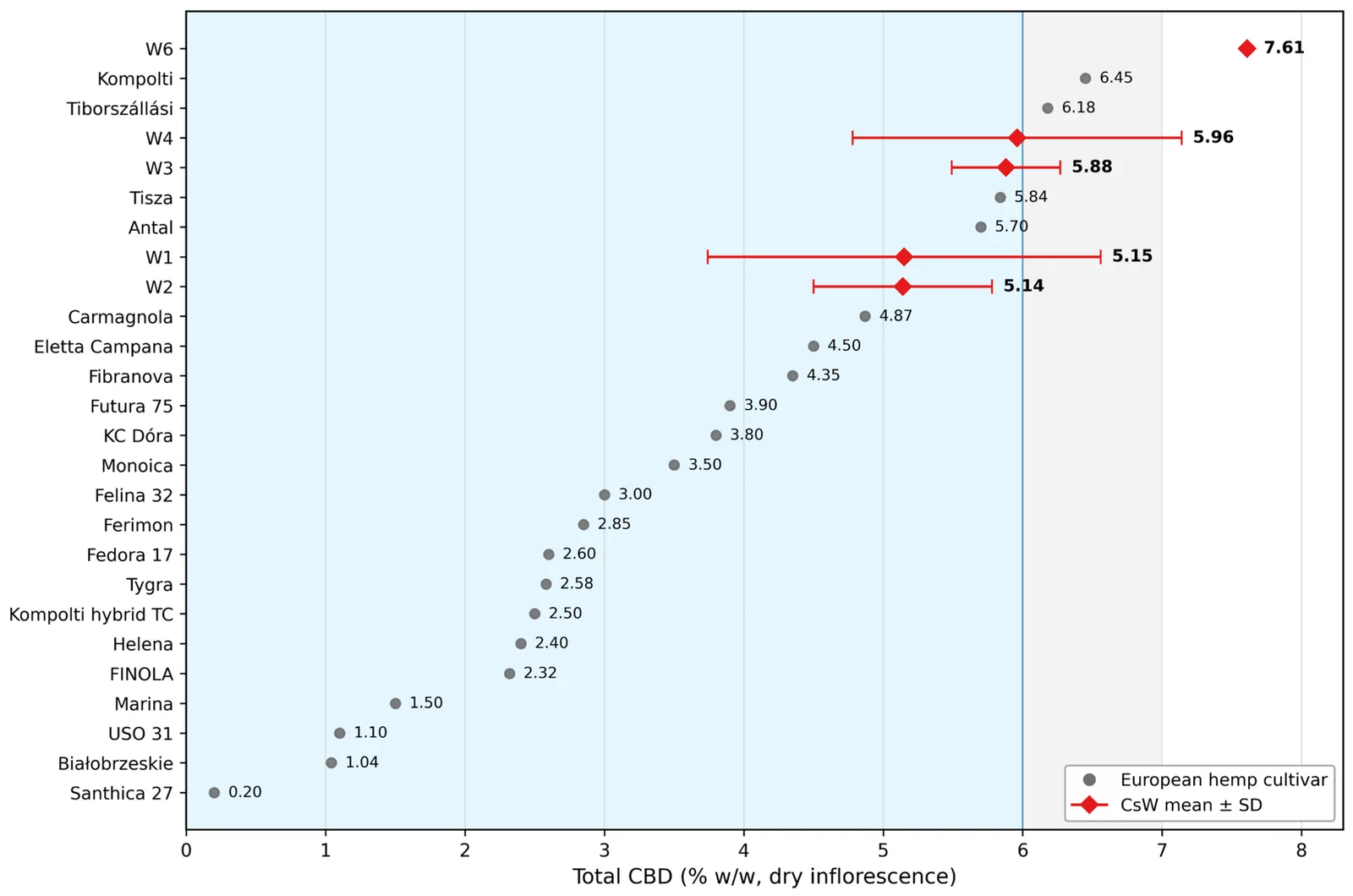
CBD landscape of European hemp cultivars and CsW. Abbreviations: CBD—cannabidiol; W1–W6—cultivated Macedonian *Cannabis sativa* plant groups of wild-origin (CsW).

The cannabinoid profile of the CsW was also compared with published inflorescence data for European hemp cultivars (Figure 3 and Supplementary Table S7). A clear positioning of CsW within the upper part of the reported CBD range was observed. Mean total CBD concentrations of W1–W4 ranged from 5.14 to 5.96%, placing these groups alongside or above several established CBD-dominant cultivars, whereas W6-2 reached 7.61%, representing the highest value observed within the present material. In the literature dataset, cultivars such as Fedora 17, Felina 32, FINOLA, Futura 75, USO 31, Kompolti hybrid TC and Carmagnola generally showed lower total CBD concentrations under the reported experimental conditions, while higher-CBD cultivars such as Antal, Tisza, Tiborszállási and Kompolti approached the range observed for W1–W4 [11,12,27,28,29,30,31,32,33]. Importantly, the relevance of the CsW is not based solely on relatively high CBD accumulation, but on its combination of CBD predominance and low total Δ9-THC, with mean THC values of only 0.14–0.17% in W1–W4. This phytochemical balance is particularly noteworthy when contrasted with Charlotte’s Angel, which accumulated higher CBD concentrations under the same cultivation conditions but also exceeded the low-THC threshold. Although direct quantitative comparisons among studies should be interpreted cautiously because cannabinoid accumulation is strongly influenced by genotype, environment, developmental stage, cultivation practices and analytical methodology, the overall positioning shown in Figure 3 indicates that CsW combine CBD levels characteristic of the higher-CBD segment of European hemp with comparatively low THC content. This profile further supports their characteristics and their potential for agronomic characterization, phytochemical selection, and evaluation in future hemp breeding and registration-oriented programs.

**Figure 4:**
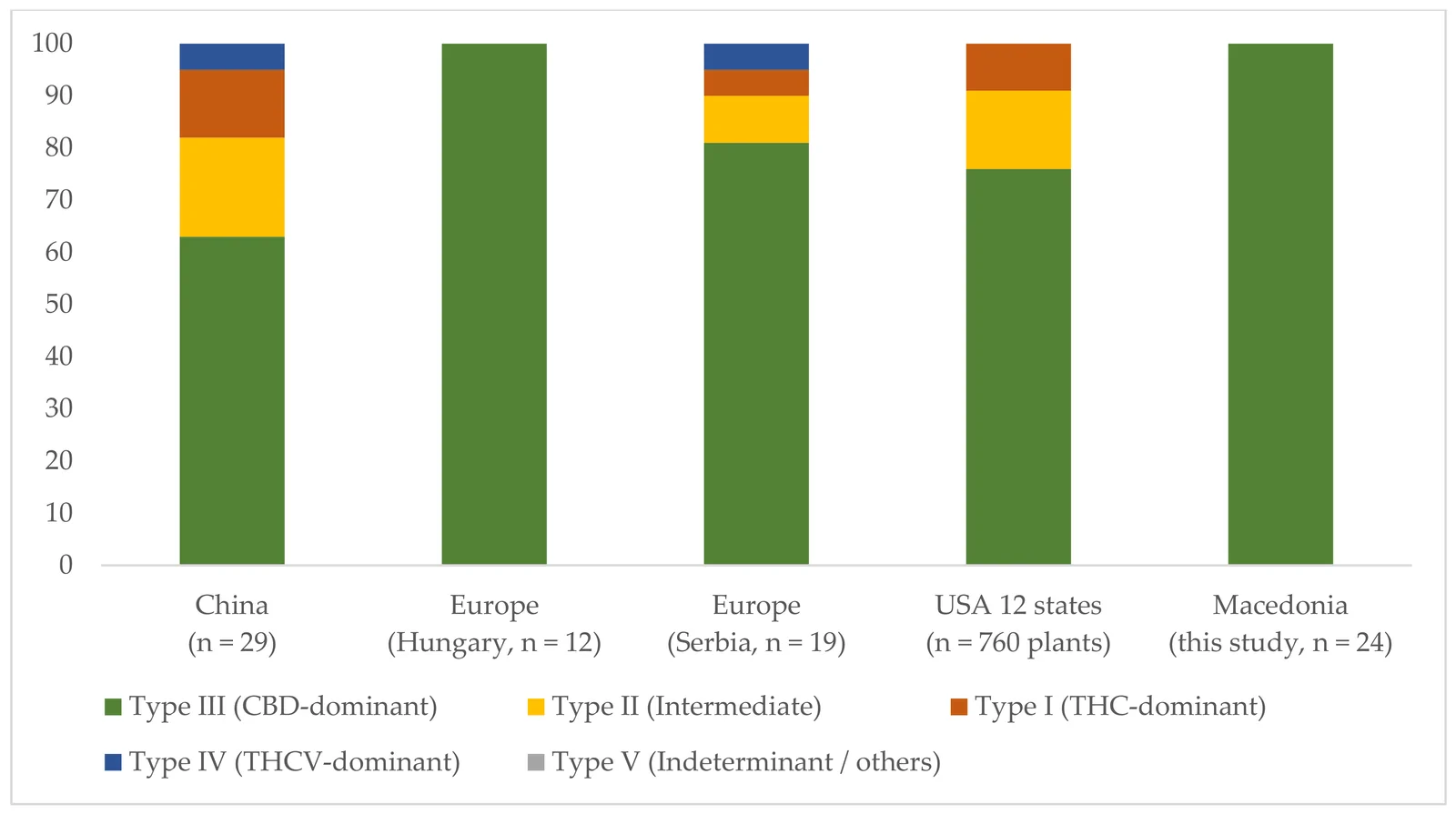
Chemotype [25] (cannabinoid profile) distribution of wild cannabis populations from different geographical origins.

Comparison with wild and feral cannabis from other geographical origins further contextualized the relatively homogeneous cannabinoid profile of the CsW (Figure 4). In contrast, Mehdi et al. [34] reported pronounced chemotypic heterogeneity among 66 naturally occurring Indian populations, of which 45 were characterized by THC > 0.3% and CBD < 0.5%, whereas only five showed THC < 0.3% and CBD > 0.5%. Feral populations from North America similarly showed a predominance of CBD-rich/Type III plants, although Type I and Type II chemotypes were also present [25], indicating greater chemotypic heterogeneity [35,36]. Notably, total cannabinoid content in US feral material ranged from 0.21–4.73%, whereas total CBD alone reached 5.14–7.61% in the CsW, although differences in cultivation, sampling and analytical methodology preclude direct quantitative comparison [30]. Reports of spontaneous or wild cannabis from Hungary and Serbia further demonstrate the persistence of phytochemically and morphologically distinct populations in Central and Southeastern Europe [37,38,39]. Comparable population-level cannabinoid data for wild or feral African cannabis remain scarce, with the chemical diversity of southern African germplasm still insufficiently characterized [40,41].

### 2.3. Terpene Profile

**Figure 5:**
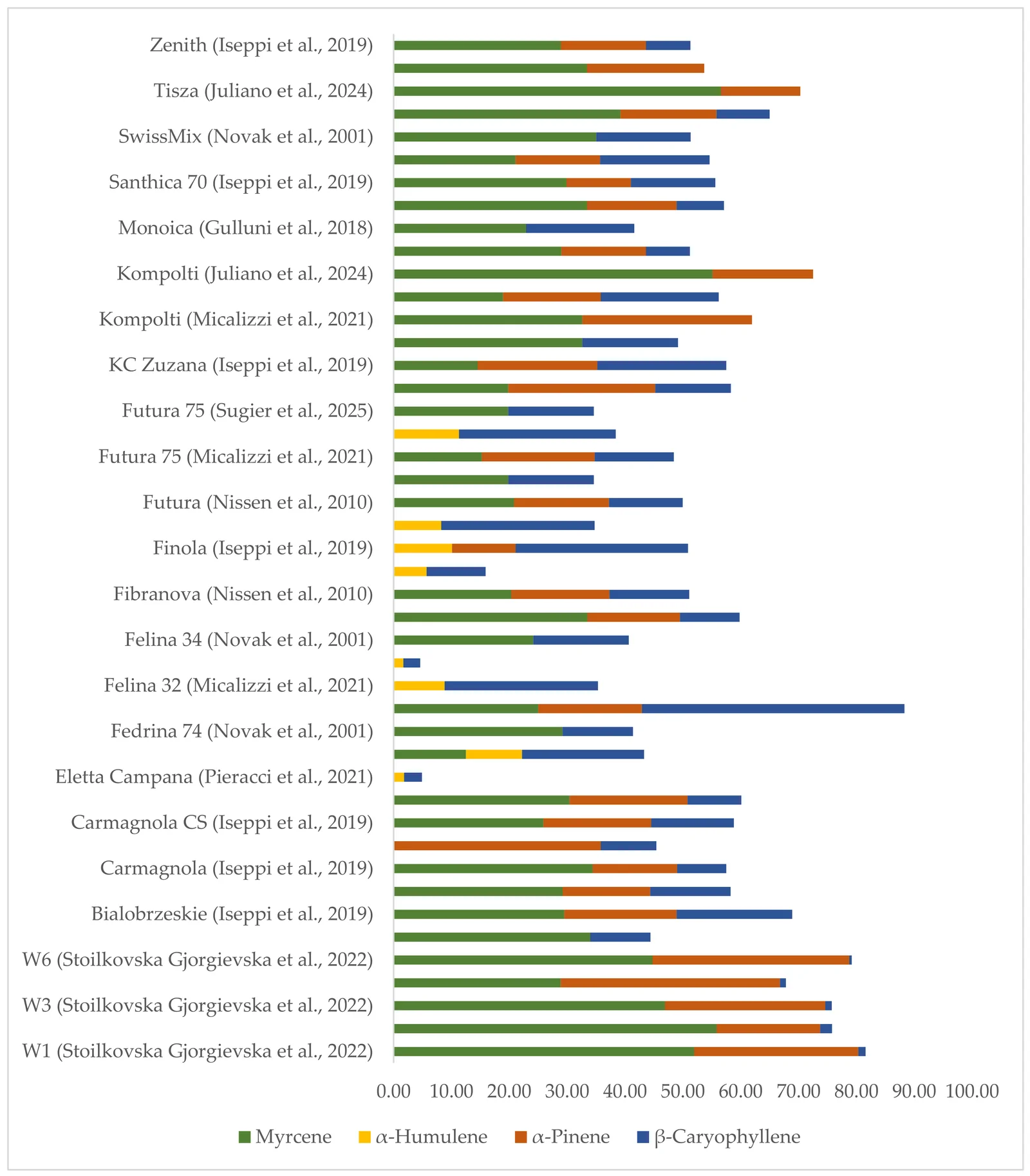
Comparison of the relative abundance (%) of selected predominant terpenes (myrcene, α-humulene, α-pinene, and β-caryophyllene) in CsW and several European hemp cultivars reported in the literature [46,49,50,51,52,53,54,55,56,57,58,59,60]. Abbreviations: W1–W6—cultivated Macedonian *Cannabis sativa* plant groups of wild-origin (CsW).

Terpene composition has increasingly been considered as an additional criterion for the chemical differentiation of cannabis, particularly in chemovar classification based on major cannabinoids and dominant terpenes [42]. Moreover, variation in the relative abundance of terpenes among cannabis chemotypes further supports their value as complementary phytochemical characteristics [43,44,45]. As part of the broader phytochemical characterization and positioning of the CsW, the previously reported terpene profiles, [46,47] analyzed with HS-GS/MS/FID [48], were further contextualized using published data for registered hemp varieties (Figure 5).

Overall, the CsW showed a terpene pattern consistent with the chemical diversity reported for European hemp, with myrcene and α-pinene representing the principal constituents [47]. Myrcene has also been reported as a major or predominant terpene in several European hemp cultivars, including Bialobrzeskie, Carmagnola, Ferimon, Futura 75, Santhica 27, Santhica 70, Tiborszallasi and Tygra, although the relative contributions of α-pinene, β-caryophyllene and other terpenes vary considerably among cultivars and studies. Particularly close qualitative agreement was observed with Kompolti and Tisza, for which Juliano et al. (2024) reported myrcene as the predominant constituent (55.10 and 56.56%, respectively), followed by α-pinene (17.40 and 13.73%) and limonene (10.31 and 6.53%) [49]. Earlier data for Kompolti also demonstrated a myrcene-rich profile, although with greater contributions from caryophyllene and ocimene [59], highlighting the substantial influence of growing conditions, developmental stage, plant material and analytical approach on the relative terpene composition. Other cultivars, such as Felina 32 and Fedora 17, have been reported to exhibit more pronounced β-caryophyllene-dominant profiles, further demonstrating the chemotypic variability of European hemp. Taken together, the predominance of myrcene and α-pinene positions the CsW within the broader terpene diversity reported for European hemp varieties, while group-specific differences in the relative contributions of these and other major terpenes further demonstrate the phytochemical distinctiveness of the investigated material.

## 3. Materials and Methods

### 3.1. Plant Material

Plants and seeds from wild-origin *Cannabis sativa* L. were collected during the flowering and seed maturation stages in July 2019 and August 2020 from several localities in the Kočani and Strumica regions of North Macedonia. Plant identification was performed by Prof. Gjoshe Stefkov (Faculty of Pharmacy, Ss. Cyril and Methodius University in Skopje, North Macedonia) based on characteristic morphological features and selected descriptors of the UPOV guideline TG/276/1 for *Cannabis sativa* L. A complete overview of all identified plants from wild-origin cannabis, including collection sites, geographical coordinates and herbarium voucher specimens deposited at the Herbarium of the Faculty of Pharmacy (FFUKIMCs), and is published in our previous research [17,61].

Five groups of plants of wild-origin from Kočani and Strumica regions were selected for subsequent outdoor cultivation and comprehensive characterization (Table 3).

Four commercial cannabis cultivars with contrasting cannabinoid chemotypes were included in the study, namely Charlotte’s Angel (CA; chemotype III class, CBD-predominant), Bubba Kush × OG Kush [26] (BK × OGK; chemotype I, Δ9-THC-predominant), NYC Diesel (NYCD; chemotype I, Δ9-THC-predominant) and Great White Shark (GWS; chemotype II, intermediate Δ9-THC/CBD ratio). Voucher specimens of cultivated Macedonian *Cannabis sativa* plants of wild-origin (CsW) and commercial cultivars were deposited at the Herbarium of the Faculty of Pharmacy.

For each CsW, the seed lot originated from a single maternal plant collected at the corresponding natural locality; seeds collected from different maternal plants at the same locality were not pooled. Following germination, the resulting plants were cultivated separately according to their respective seed lots. Germination percentages and the numbers of plants established for cultivation, together with their sex distribution, is summarized in Supplementary Table S4. All plants identified as male during the vegetative stage or at the beginning of the generative stage were removed from the experimental field to prevent cross-pollination with other cannabis hybrids cultivated by the company. Identification of male and female flowers is shown in Supplementary Figures S4 and S5. Two plants (BK × OGK-6 and NYCD-3) were lost during field cultivation due to mechanical stem breakage.

### 3.2. Seed Germination and Cultivation

Seeds collected from Macedonian *Cannabis sativa* plants of wild-origin and seeds of commercial cultivars were subjected to germination trials in order to obtain plant material for subsequent morphological and phytochemical characterization. Preliminary experiments evaluated seed soaking and mechanical scarification under moist conditions, with the latter approach proving more suitable for plants from wild-origin cannabis seeds. Prior to germination, seeds were mechanically scarified using P80 abrasive paper and placed between two layers of moistened cotton pads in Petri dishes. Petri dishes were incubated in darkness at approximately 25 °C, and germination was monitored daily. Germinated seeds were carefully transferred into seedling trays containing commercial propagation substrate. Germination percentage was calculated as the ratio between the number of germinated and tested seeds. Germination percentages for all plant groups are presented in Supplementary Table S4.

**Figure A1:**
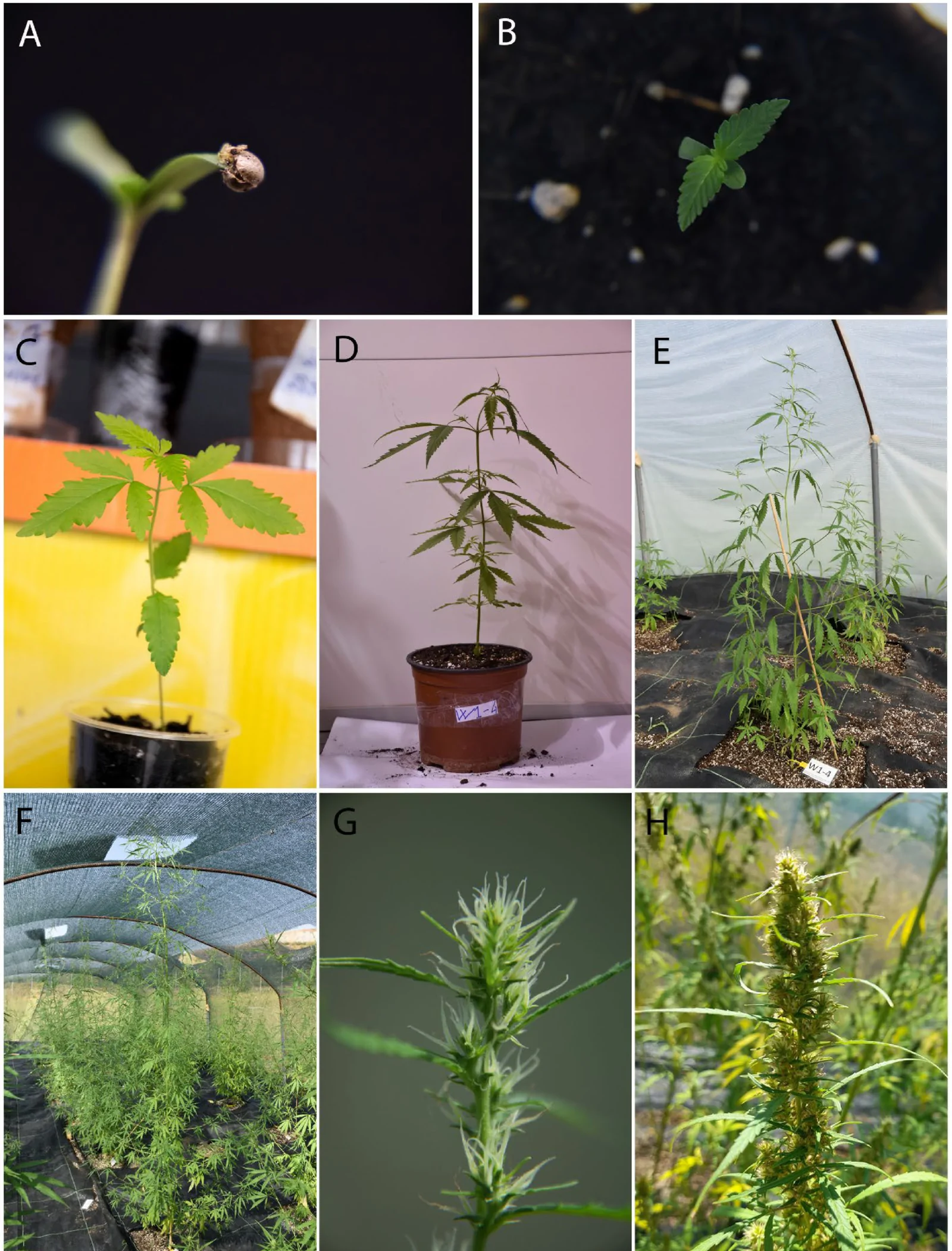
Stages of the cultivation process of plants from the W1 group (Gorni and Dolni Podlog, Kočani region): (**A**) emergence with the seed coat still attached, (**B**) first two true leaves, (**C**) early vegetative development, (**D**) early vegetative development and preparation for transfer to outdoor conditions, (**E**) late vegetative development, (**F**) onset of flowering, (**G**) flower appearance at the onset of flowering, and (**H**) later flowering stage.

Seedlings were initially maintained under controlled indoor conditions under an 18 h light/6 h dark photoperiod at ambient temperature. During the vegetative stage, plants were fertilized with a water-soluble NPK fertilizer enriched with micronutrients—Alkafert, N:P:K 10:5:5 supplemented with Cu, Mn and Zn (Alkaloid AD, Skopje, North Macedonia), applied at a concentration of 0.3% according to the manufacturer’s recommendations. Prior to transplantation to the outdoor experimental field, plants were acclimatized under greenhouse conditions for approximately one month. Outdoor cultivation was conducted during the 2021 growing season at the facilities of a licensed cannabis company (North Macedonia). Plants were cultivated in a substrate consisting of equal volumes of local field soil, organic compost and commercial growing substrate (1:1:1, *v*/*v*/*v*). During cultivation, plants received periodic applications of a liquid NPK fertilizer supplemented with magnesium (20:20:20 + Mg), whereas copper fertilizer containing 14% Cu was used to reduce the risk of bacterial and fungal infections. Insecticide treatments based on flonicamid (500 ± 25 g/L) (ISK Biosciences Europe N.V., Diegem, Belgium) were applied when required to minimize biotic stress. Plants were grown under insect netting and shading nets (40–60%) and irrigated regularly to maintain adequate soil moisture. Female inflorescences were harvested at technological maturity, manually cleaned and air-dried under dark, well-ventilated conditions for seven days. Dried plant material was subsequently packed in labelled aluminum bags and stored until further morphological and phytochemical analyses. The cultivation process is presented in Figure A1 and Supplementary Figure S7.

### 3.3. Morphological Characterization

Morphological characterization of CsW and commercial cultivars was performed according to the UPOV guideline TG/276/1 for *Cannabis sativa* L. A total of 18 qualitative and quantitative descriptors related to plant architecture, foliage, inflorescence morphology, and phenology were evaluated and recorded as states of expression. Descriptive observations and measurements were conducted throughout the vegetative and reproductive developmental stages of female plants cultivated under uniform outdoor conditions. Seed material originating from the investigated plants of wild-origin and commercial cultivars was previously subjected to detailed morphometric characterization by digital image analysis, as described by Stoilkovska Gjorgievska et al. [17].

Because plant breeders’ rights for cannabis varieties are protected under the UPOV Convention, detailed morphological descriptions generated during variety registration and DUS testing are often not publicly accessible, which limits the availability of reference data for comparative studies. Among varieties registered by companies from European Union Member States, Estica, registered in Estonia, was identified as the only variety for which a sufficiently detailed UPOV-based morphological description was publicly available [62]. In contrast, Australia maintains a governmental *Plant Breeder’s Rights* database in which morphological characteristics and examination data for registered plant varieties, including cannabis, are publicly documented in the *Plant Varieties Journal*, issued four times annually [22]. Therefore, to compare the cultivated material examined in this study with officially registered cannabis varieties, the publicly available UPOV-based description of Estica, together with those of varieties documented within the Australian *Plant Breeder’s Rights* system, was incorporated into the multivariate analyses. This approach enabled the assessment of phenotypic similarities among CsW, commercial cultivars, and officially registered cannabis varieties and may provide useful guidance for selecting appropriate reference varieties for future distinctness, uniformity and stability (DUS) testing procedures.

### 3.4. HPLC-DAD Analysis of Cannabinoids

Cannabinoid analysis was performed according to the monograph for *Cannabis flos* of the German Pharmacopoeia (DAB) with minor modifications [63,64]. Dried female inflorescences were milled and sieved through a 710 μm sieve. Approximately 0.5 g of powdered plant material was extracted with 20 mL of 96% ethanol (Ph. Eur., Alkaloid AD, Skopje, North Macedonia) by ultrasonication (50/60 Hz) for 15 min and subsequently centrifuged. The residue was extracted twice more with 12.5 mL aliquots of ethanol under identical conditions. Combined extracts were transferred into a 50 mL volumetric flask and adjusted to volume with 96% ethanol. Prior to analysis, samples were filtered through regenerated cellulose membrane filters (0.45 μm). Subsequently, 1 mL of filtrate was transferred into a 10 mL volumetric flask and diluted to volume with 96% ethanol to obtain a final concentration of 1 mg/mL. All samples were prepared in triplicate.

Chromatographic analysis was performed using an Agilent 1200 HPLC system equipped with a quaternary pump (G1311A), thermostatted autosampler (G1329A), column compartment (G1316A) and diode array detector (Agilent Technologies, Waldbrune, Germany). Separation was achieved on an InfinityLab Poroshell 120 EC-C18 column (150 × 3 mm, 2.7 μm; Agilent Technologies, Santa Clara, CA, USA) maintained at 40 °C. The mobile phase consisted of aqueous orthophosphoric acid solution (8.64 g/L) as solvent A and HPLC-grade acetonitrile (Carlo Erba Reagents S.r.l., Cornaredo, Italy) as solvent B. Gradient elution was performed according to the following program: 0–16 min, 36–18% A; 16–17 min, 18–36% A; followed by a re-equilibration period up to 30 min. The flow rate was maintained at 0.7 mL/min, the injection volume was 5 μL, and chromatograms were recorded at 225 nm.

Quantification was performed by external calibration using certified reference standards of CBDA, CBD, CBN, Δ9-THC and Δ9-THCA (Cayman Chemical, Ann Arbor, MI, USA). Calibration curves were constructed according to DAB requirements using at least five concentration levels. The calibration ranges were 0.5–100 μg/mL for CBDA, 0.5–75 μg/mL for CBD and THC, 0.1–10 μg/mL for CBN, and 0.5–250 μg/mL for Δ9-THCA.

System suitability was evaluated according to DAB requirements. Resolution between Δ9-THC and Δ8-THC peaks was required to be not less than 1.2, whereas precision was assessed by six replicate injections of the Δ9-THCA reference solution (50 μg/mL), with an acceptance criterion of relative standard deviation not exceeding 1.0%.

### 3.5. Chemometric Analysis

Principal component analysis (PCA), hierarchical cluster analysis (HCA), and partial least squares discriminant analysis (PLS-DA) were performed using SIMCA v14.1 software. Model performance was evaluated using R^2^X, R^2^Y, and Q^2^ parameters. Variable importance in projection (VIP) values and loading plots were used to identify descriptors contributing most strongly to sample discrimination. HCA was performed using Ward’s linkage method following min–max normalization (0–1 scaling). PLS-DA was subsequently applied to the HCA-derived groups as an exploratory supervised step to further characterize and visualize the group structure and to identify the variables contributing to sample differentiation, rather than as an independent classification model. Internal model validation was performed by 50-permutation testing and CV-ANOVA. The experimental morphology, expanded UPOV, and cannabinoid PLS-DA models comprised 55, 48, and 50 observations, respectively, with 19 morphological/UPOV descriptors, 28 UPOV descriptors, and nine cannabinoid-related variables, respectively. Morphological descriptors, cannabinoid contents and publicly available UPOV datasets of registered cannabis varieties were integrated into the multivariate analyses to investigate phenotypic and phytochemical relationships among CsW, commercial cultivars, and registered cannabis varieties.

## 4. Conclusions

This study demonstrates that Macedonian *Cannabis sativa* plants of wild-origin, after cultivation under common outdoor conditions, retain a distinct and structured morphological and phytochemical profile. UPOV-based multivariate analyses clearly separated the plants of wild-origin from the commercial cultivars and revealed additional differentiation associated with geographical origin. Most plants of wild-origin also showed morphological proximity to Kompolti, indicating phenotypic similarities with this established hemp-type variety and suggesting its potential relevance as a comparator in future DUS-oriented evaluation.

All CsW were CBD-predominant, with total CBD of 5.14–7.61% and total Δ9-THC at or below 0.20%. The fusion of morphological character, favorable agronomic performance, relatively high CBD accumulation and consistently low Δ9-THC highlights these plants as a potentially valuable resource for future hemp selection and development. The present findings provide a strong basis for further evaluation of the most promising wild-origin plant groups but broader multi-environment assessment would further establish the stability of their favorable characteristics and their adaptability to different growing conditions. Complementary genetic characterization could additionally support the observed morphological and phytochemical differentiation.

Further selection, stability assessment and multi-environment evaluation are necessary to determine its potential for breeding and future variety development. Overall, the integration of morphological, agronomic and cannabinoid characteristics identifies Macedonian wild-growing *Cannabis sativa* as a valuable and still insufficiently explored resource for hemp improvement. The identified CBD-rich and low-THC populations provide promising starting material for targeted selection, with potential application in the development of hemp varieties and future DUS-oriented evaluation and breeding programs.

## Figures and Tables

**Table 1:** Summary of morphological and agronomic characteristics of cultivated Macedonian *Cannabis sativa* plants of wild-origin and commercial cannabis strains.

	Plant Group	W1	W2	W3	W4	W6	CA	BK × OGK	NYCD	GWS
Descriptor										
**Cotyledon shape**		Narrow–medium obovate	Narrow obovate	Narrow obovate	Narrow obovate	Narrow obovate	Broad obovate	Narrow obovate	Narrow obovate	Narrow–medium obovate
**Cotyledon color**		Medium–dark green	Medium–dark green	Medium–dark green	Medium–dark green	Medium–dark green	Light–medium green	Dark green	Light green	Light green
**Hypocotyl anthocyanin coloration**		Medium	Weak	Weak–strong	Weak	Weak	Weak	Weak	Very strong	Weak–strong
**Crown anthocyanin coloration**		Absent/very weak	Absent/very weak	Absent/very weak	Absent/very weak	Absent/very weak	Absent/very weak	Absent/very weak	Absent/very weak	Absent/very weak
**Leaf green-color intensity**		Medium	Medium	Medium	Medium	Medium	Medium	Medium–dark	Medium	Medium
**Number of leaflets**		Few (3–5)	Few (3–5)	Few (3–5)	Few (3–5)	Few (3–5)	Few (5)	Few–medium (5–7)	Few–medium (5–7)	Few–medium (5–7)
**Main stem color**		Yellow	Yellow	Yellow	Yellow	Yellow	Yellow	Yellow	Green	Green
**Main stem thickness**		Medium–thick	Medium–thick	Medium–thick	Medium–thick	Medium–thick	Thick	Thick	Thick	Thick
**Depth of stem grooves**		Shallow	Shallow	Shallow	Shallow	Shallow	Shallow–medium	Medium	Medium–deep	Medium
**Pith appearance in cross-section**		Thick	Thick	Thick	Thick	Thick	Thick	Thick	Thick	Thick
**Proportion of female plants (Female:Male)**		10:1	3:3	4:3	6:5	1:1	Female only	Female only	Female only	Female only
**Central leaflet length (mm), mean ± SD**		34.71 ± 6.63	45.43 ± 8.25	28.52 ± 4.05	40.58 ± 8.27	—	57.87 ± 5.90	25.15 ± 3.63	27.40 ± 9.37	27.68 ± 6.28
**Central leaflet width (mm), mean ± SD**		1.86 ± 0.45	1.95 ± 0.75	0.97 ± 0.14	1.77 ± 0.79	—	4.22 ± 1.04	1.79 ± 0.68	1.48 ± 0.62	1.39 ± 0.58
**Plant height (cm), mean ± SD**		213.40 ± 17.04	226.33 ± 25.11	235.25 ± 17.15	230.83 ± 13.99	—	183.67 ± 18.98	145.25 ± 30.97	122.75 ± 9.54	149.00 ± 12.00
**Untrimmed flower yield (g), mean ± SD**		84.43 ± 11.83	89.46 ± 2.88	73.96 ± 8.79	71.79 ± 17.35	—	92.67 ± 22.60	59.36 ± 26.13	66.80 ± 29.11	56.75 ± 12.31

Abbreviations: W1–W6—cultivated Macedonian *Cannabis sativa* plant groups of wild-origin (CsW); CA—Charlotte’s Angel; BK × OGK—Bubba Kush × OG Kush; GWS—Great White Shark; NYC Diesel—New York City Diesel; SD—standard deviation; (—)—not calculated, mm—millimeter, cm—centimeter.

**Table 2:** UPOV morphological descriptors identified from the loading/weighting plots as the main contributors to the separation of HCA-defined groups in the PLS-DA model based on morphological characterization of the investigated cannabis material.

Principal Component	HCA Groups	Statistically Significant Morphological Characteristics Differentiating the Groups
PC1 (20.97%)	Group 1 (predominantly Kočani region) and Group 2 (predominantly Strumica region)	▪ Cotyledon: color (green to dark green) ▪ Leaf: number of leaflets (3–5) ▪ Main stem: thickness (medium to thick) ▪ Main stem: depth of grooves (shallow) ▪ Seed: color of testa (brown) ▪ Seed: marbling (strong) ▪ Plant: height
	Group 3 (Charlotte’s Angel), Group 4 (Bubba Kush × OG Kush), Group 5 (NYC Diesel) and Group 6 (Great White Shark)	▪ Leaf: number of leaflets (5) ▪ Plant: proportion of female plants (female plants only) ▪ Main stem: thickness (thick)
PC2 (19.88%)	Group 3 (Charlotte’s Angel)	▪ Cotyledon: shape (broad ovate) ▪ Cotyledon: color (light green to green) ▪ Leaf: length of central leaflet
	Groups 4 (Bubba Kush × OG Kush), 5 (NYC Diesel) and 6 (Great White Shark)	▪ Cotyledon: shape (narrow ovate) ▪ Leaf: number of leaflets (few—5 to medium—7)
PC3 (18.65%)	Group 4 (Bubba Kush × OG Kush)	▪ Hypocotyl: intensity of anthocyanin coloration (weak) ▪ Main stem: color (yellow)
	Groups 5 (NYC Diesel) and 6 (Great White Shark)	▪ Cotyledon: color (light green) ▪ Main stem: color (green) ▪ Main stem: depth of grooves (medium to deep) ▪ Seed: color of testa (grey brown) ▪ Seed: marbling (absent)
PC4 (21.25%)	Group 1 (predominantly Kočani region)	▪ Cotyledon: shape (narrow to medium ovate) ▪ Hypocotyl: intensity of anthocyanin coloration (medium) ▪ Plant: proportion of female plants (high)
	Group 2 (predominantly Strumica region)	▪ Cotyledon: shape (narrow ovate) ▪ Hypocotyl: intensity of anthocyanin coloration (weak) ▪ Plant: proportion of female plants (medium)
PC5 (17.34%)	Group 5 (NYC Diesel)	▪ Hypocotyl: intensity of anthocyanin coloration (weak to medium) ▪ Leaf: number of leaflets (few—5) ▪ Main stem: depth of grooves (medium)
	Group 6 (Great White Shark)	▪ Hypocotyl: intensity of anthocyanin coloration (very strong) ▪ Leaf: number of leaflets (few—5 to medium—7) ▪ Main stem: depth of grooves (medium to deep)

**Table 3:** Collection localities and year of collection of *cannabis* plants of wild-origin.

Cultivated Plant Group	Region	Locality Where Cannabis Plants Were Identified and Collected in Seeding Stage/Year of Collection
W1	Kočani	Road between the villages of Gorni Podlog and Dolni Podlog/2019
W2	Kočani	Village Dolni Balvan/2020
W3	Strumica	Between the villages of Prosenikovo and Saraj, along the Turija River (on both sides of the river)/2019
W4	Strumica	Bridge near the village of Bosilovo, on the left bank downstream of the Strumica River/2019
W6	Kočani	Village Mojanci/2020

## Data Availability

The data presented in this study are available within the article and its Supplementary Materials. Additional data supporting the findings of this study will be made available by the authors on request.

## Supplementary Materials

The following supporting information can be downloaded at https://www.mdpi.com/article/10.3390/molecules31203723/s1. Table S1: Quantitative characterization of leaf traits and plant height according to UPOV descriptors, and untrimmed flower yield of individual plants of outdoor-cultivated (CsW) and commercial strains; Table S2: Quantitative characterization of leaf traits and plant height according to UPOV descriptors, and untrimmed flower yield in CsW and commercial strain plant groups; Table S3: CV-ANOVA results for the PLS-DA models; Table S4: Germination and sex distribution of the cultivated *Cannabis sativa* plant groups; Table S5: Cannabinoid content in dried flowers of individual CsW from groups W1, W2, W3, W4, and W6 (expressed as % m/m); Table S6: Cannabinoid content in dried flowers of individual cultivated commercial plants (CA, NYCD, and GWS), expressed as % m/m; Table S7: Reported total CBD content in inflorescences of selected European hemp cultivars and CsW; Figure S1: Permutation test (*n* = 50) for the morphology-based PLS-DA model. The model comprised six components and yielded R^2^ and Q^2^ intercepts of 0.0491 and −0.509, respectively; Figure S2: Permutation test (*n* = 50) for the expanded UPOV morphology -based PLS-DA model. The model comprised three components and yielded R^2^ and Q^2^ intercepts of 0.409 and −0.291, respectively; Figure S3: Permutation test (*n* = 50) for the cannabinoid composition based PLS-DA model. The model comprised three components and yielded R^2^ and Q^2^ intercepts of 0.003 and −0.271, respectively; Figure S4: Identification of male plants: (A–C) characteristic morphological features of male plants—racemose inflorescences (inflorescentia racemosa) consisting of multiple small flowers arranged in clusters, with stamens that produce pollen; Figure S5: Identification of female plants: (A–C) Characteristic morphological features of female plants—initial flowers developing in the leaf axils (axillae), with pistils bearing characteristically long, hair-like stigmas that capture pollen for fertilization; Figure S6: PLS-DA loading plots based on the cannabinoid profiles of cultivated Macedonian *Cannabis sativa* plants from wild-origin (CsW) and commercial cannabis strains, showing the contribution of individual cannabinoids to the observed sample differentiation. Abbreviations: PC—principal component; PLS-DA—partial least square discriminatory analysis; CBDA—cannabidiolic acid; CBD—cannabidiol; CBN—cannabinol; Δ9-THC—Δ9-tetrahydrocannabinol; Δ9-THCA—Δ9-tetrahydrocannabinolic acid; AVE—average.; Figure S7: Cultivation process: (a) germination, (b) imbibition, (c) early vegetative stage, (d) preparation of the experimental field for outdoor transplantation, (e) transplanted plants at the experimental field during the early vegetative stage, (f,g) spatial arrangement of the outdoor experimental field, (h) onset of flowering, (i) harvesting, (j) drying, and (k) weighing and packaging of the dried plant material.
