# Supplementary material for: From Wild-Growing Plants to Promising Candidates for Hemp Variety Development: Morphological and Phytochemical Characterization of Macedonian Cannabis sativa L
Source: Molecules. 2026 Oct 16;31(20):3723. doi: 10.3390/molecules31203723 (PMC13707986; doi:10.3390/molecules31203723)
Supplement: Supplementary file 1 [file molecules-31-03723-s001.zip › molecules-4598894-supplementary.pdf]

Table S1. Quantitative characterization of leaf traits and plant height according to UPOV descriptors, and untrimmed flower yield of individual plants of outdoor- cultivated (CsW) and commercial strains.

Individual plant code	Leaf: length of central leaflet (mm)	Leaf: width of central leaflet (mm)	Plant height (cm)	Untrimmed flower yield (g)
W1-1	33.75	1.97	208	62.60
W1-2	49.37	2.74	200	104.73
W1-4	33.93	1.82	235	81.41
W1-5	39.48	1.48	215	83.50
W1-6	38.57	2.32	190	80.39
W1-7	27.80	1.81	190	97.01
W1-8	26.21	2.01	218	80.32
W1-9	32.31	1.35	224	76.79
W1-10	30.69	1.83	214	82.71
W1-11	35.02	1.23	240	94.84
W2-1	37.82	1.72	250	90.07
W2-3	54.20	2.79	200	86.32
W2-4	44.26	1.35	229	91.99
W3-2	28.52	0.97	223	78.42
W3-5	34.24	0.94	250	76.57
W3-6	25.65	0.82	250	79.91
W3-7	25.66	1.16	218	60.93
W4-1	52.15	2.96	227	86.75
W4-2	45.80	2.25	223	76.23
W4-3	37.24	1.98	210	45.44
W4-10	43.83	1.44	240	82.20
W4-11	35.40	1.12	235	55.01
W4-12	29.06	0.84	250	85.09
Wuc	15.80	1.48	265	100.00
W6-2	42.87	1.75	231	39.94
CA – 1	67.90	6.30	135	116.00
CA – 2	54.84	3.77	185	78.00
CA – 3	52.92	3.08	190	129.00
CA – 4	53.46	3.76	190	108.00
CA – 5	60.48	4.66	198	84.00
CA – 6	51.67	3.53	188	98.90
CA – 7	53.15	5.41	180	92.00
CA – 8	62.93	3.66	193	67.10
CA – 9	63.51	3.82	194	61.00

Individual plant code	Leaf: length of central leaflet (mm)	Leaf: width of central leaflet (mm)	Plant height (cm)	Untrimmed flower yield (g)
BKxOGK-1	28.03	3.14	130	59.70
BKxOGK-2	Not measured.		120	22.50
BKxOGK-3	29.74	1.93	141	72.20
BKxOGK-4	25.95	1.28	201	49.60
BKxOGK-5	23.91	1.23	122	96.00
BKxOGK-7	27.30	1.80	187	48.90
BKxOGK-8	20.05	1.20	131	92.00
BKxOGK-9	21.05	1.95	130	34.00
NYCD-1	41.32	2.12	133	53.00
NYCD-2	23.89	1.02	123	40.60
NYCD-4	20.90	0.89	125	66.00
NYCD-5	23.50	1.89	110	107.60
GWS-1	20.94	0.87	140	108.00
GWS-2	27.02	0.92	148	72.00
GWS-3	26.65	1.73	136	43.00
GWS-4	Not measured.		166	52.00
GWS-5	36.12	2.03	155	60.00

Abbreviations: W1- W6 - cultivated Macedonian *Cannabis sativa* plant groups from wild-origin (CsW); CA – Charlotte's Angel; BKxOGK – Bubba Kush x OG Kush; GWS – Great White Shark; NYC Diesel – New York City Diesel; mm - millimeter; cm – centimeter; g- gram.

Table S2. Quantitative characterization of leaf traits and plant height according to UPOV descriptors, and untrimmed flower yield in CsW and commercial strain plant groups

**Plant group**	Leaf: length of central leaflet (mm)			Leaf: width of central leaflet (mm)			**Plant height (cm)**			**Untrimmed flower yield (g)**		
	AVE	STD	RSD	AVE	STD	RSD	AVE	STD	RSD	AVE	STD	RSD
W1	34.71	6.63	19.11	1.86	0.45	24.27	213.40	17.04	7.99	84.43	11.83	14.01
W2	45.43	8.25	18.17	1.95	0.75	38.28	226.33	25.11	11.01	89.46	2.88	3.22
W3	28.52	4.05	14.19	0.97	0.14	14.48	235.25	17.15	7.29	73.96	8.79	11.89
W4	40.58	8.27	20.39	1.77	0.79	44.52	230.83	13.99	6.06	71.79	17.35	24.17
CA	57.87	5.90	10.20	4.22	1.04	24.56	183.67	18.98	10.33	92.67	22.60	24.39
BKx-OGK	25.15	3.63	14.43	1.79	0.68	38.09	145.25	30.97	21.32	59.36	26.13	44.02
NYCD	27.40	9.37	34.20	1.48	0.62	41.60	122.75	9.54	7.77	66.80	29.11	45.38
GWS	27.68	6.28	22.67	1.39	0.58	41.95	149.00	12.00	8.05	56.75	12.31	21.70

Abbreviations: W1- W6 - cultivated Macedonian Cannabis sativa plant groups from wild-origin (CsW); CA – Charlotte's Angel; BKxOGK - Bubba Kush x OG Kush; GWS – Great White Shark; NYC Diesel - New York City Diesel; AVE - average; STD - standard deviation; RSD - relative standard deviation.

Table S3. CV-ANOVA results for the PLS-DA models.

Dataset / model	Source	SS	DF	MS	F	*p*	SD
**Experimental morphology**	Total corr.	270	270	1	–	–	1
	Regression	256.122	60	4.2687	64.5925	<0.001	2.06608
	Residual	13.8782	210	0.0660866	–	–	0.257073
**Expanded UPOV**	Total corr.	141	141	1	–	–	1
	Regression	80.7393	18	4.48551	9.15552	2.23749×10^{-15}	2.1179
	Residual	60.2607	123	0.489925	–	–	0.699946
**Cannabinoid content**	Total corr.	98	98	1	–	–	1
	Regression	73.4513	12	6.12094	21.4431	5.92396×10^{-21}	2.47405
	Residual	24.5487	86	0.28545	–	–	0.534275

Abbreviations: CV-ANOVA - analysis of variance of cross-validated residuals; PLS-DA - partial least squares discriminant analysis; SS - sum of squares; DF - degrees of freedom; MS - mean square; SD - standard deviation.

Note: For the experimental morphology model, Simca 14.0 displayed $p = 0$; this is reported as $p < 0.001$.

Table S4. Germination and sex distribution of the cultivated *Cannabis sativa* plant groups.

Group	Germination (%)	Plants established for cultivation (n)	Male (n)	Female (n)	Notes
W1	65	11	1	10	—
W2	52	6	3	3	—
W3	23	7	3	4	—
W4	45	11	5	6	—
W5	5	1	1	0	—
W6	20	2	1	1	—
CA	100	9	0	9	—
BKxOGK	60	9	0	9	BKxOGK-6 died due to a broken stem.
NYC Diesel	100	5	0	5	NYCD-3 died due to a broken stem.
GWS	100	5	0	5	—

Abbreviations: W1- W6 - cultivated Macedonian *Cannabis sativa* plant groups from wild-origin; CA – Charlotte's Angel; BKxOGK – Bubba Kush x OG Kush; GWS – Great White Shark; NYC Diesel – New York City Diesel.

Note: Each wild-origin seed lot originated from a single maternal plant collected at the corresponding natural locality; seeds from different maternal plants at the same locality were not pooled. All plants identified as male during the vegetative stage or at the beginning of the generative stage were removed from the experimental field to prevent cross-pollination with other cannabis hybrids cultivated by the company under indoor conditions.

Table S5. Cannabinoid content in dried flowers of individual CsW from groups W1, W2, W3, W4, and W6 (expressed as % m/m).

Sample	CBDA (m/m%)	CBD (m/m%)	CBN (m/m%)	Δ9-THC (m/m%)	Δ9-THCA (m/m%)	Total CBD (m/m%)	Total Δ9-THC (m/m%)
W1-1	4.57	0.32	ND	0.02	0.11	4.33	0,11
W1-2	4.90	0.81	0.03	0.10	0.07	5.10	0.16
W1-4	3.31	0.64	ND	0.06	0.04	3.53	0.09
W1-5	7.86	0.36	ND	0.04	0.18	7.26	0.20
W1-6	4.47	0.53	BLQ	0.05	0.07	4.45	0.11
W1-7	5.81	0.87	ND	0.08	0.11	5.96	0.17
W1-8	6.82	0.61	ND	0.04	0.18	6.59	0.20
W1-9	3.85	0.67	0.01	0.10	0.05	4.04	0.14
W1-10	7.47	0.25	ND	0.03	0.16	6.80	0.17
W1-11	3.44	0.41	ND	0.01	0.05	3.43	0.06
W2-1	4.44	0.58	BLQ	0.06	0.06	4.48	0.11
W2-3	5.68	0.77	0.03	0.08	0.09	5.75	0.16
W2-4	5.75	0.16	BLQ	0.02	0.15	5.20	0,15
W3-2	6.17	0.69	BLQ	0.07	0.13	6.10	0.18
W3-5	6.08	0.14	ND	0.01	0.14	5.47	0.14
W3-6	6.22	0.85	BLQ	0.08	0.10	6.30	0.17
W3-7	5.51	0.80	BLQ	0.08	0.08	5.63	0.14
W4-1	4.59	0.43	BLQ	0.07	0.16	4.46	0.20
W4-2	7.63	0.86	BLQ	0.06	0.15	7.55	0.19
W4-3	5.75	0.52	0.01	0.04	0.11	5.57	0.13
W4-10	7.41	0.63	BLQ	0.06	0.15	7.13	0.19
W4-11	5.73	0.13	ND	0.01	0.14	5.16	0.13
W4-12	6.02	0.65	BLQ	0.07	0.10	5.93	0.15
W6-2	7.97	0.63	0.01	0.06	0.16	7.61	0.20

Abbreviations: W1- W6 - cultivated Macedonian *Cannabis sativa* plant groups from wild-origin (CsW); CBDA – cannabidiolic acid; CBD – cannabidiol; CBN – cannabinol; Δ9-THC - Δ9-tetrahydro-cannabinol; Δ9-THCA - Δ9-tetrahydrocannabinolic acid; BLQ – below limit of quantification, ND – not detected; m/m % - percentage by mass (mass/mass).

Table S6. Cannabinoid content in dried flowers of individual cultivated commercial plants (CA, NYCD, and GWS), expressed as % m/m.

Sample	CBDA (m/m%)	CBD (m/m%)	CBN (m/m%)	Δ9-THC (m/m%)	Δ9-THCA (m/m%)	Total CBD (m/m%)	Total Δ9-THC (m/m%)
CA - 1	7.15	1.05	BLQ	0.10	0.18	7.32	0.26
CA - 2	10.99	1.22	0.02	0.29	0.29	10.85	0.55
CA - 3	12.11	1.94	0.01	0.17	0.32	12.56	0.46
CA - 4	11.58	1.44	0.01	0.36	1.86	11.60	1.99
CA - 5	10.26	1.14	0.01	0.12	0.29	10.14	0.37
CA - 6	10.85	0.40	BLQ	0.05	0.38	9.91	0.38
CA - 7	9.87	3.02	0.01	0.20	0.33	11.68	0.48
CA - 8	9.38	1.48	0.01	0.15	0.24	9.70	0.36
CA - 9	12.17	2.18	0.01	0.21	0.39	12.85	0.55
NYCD-1	0.03	0.01	0.01	1.37	9.63	0.03	9.81
NYCD-2	0.02	0.01	0.01	0.83	7.94	0.03	7.80
NYCD-3	0.02	0.01	0.01	0.69	10.33	0.03	9.75
NYCD-4	0.03	0.01	0.01	1.78	11.88	0.04	12.20
GWS-1	7.62	0.09	*ND*	0.34	9.57	6.78	8.74
GWS-2	10.54	0.13	*ND*	0.23	5.66	9.37	5.19
GWS-3	2.16	0.25	0.03	1.78	10.53	2.14	11.01
GWS-4	2.36	0.20	0.01	0.51	2.22	2.27	2.45
GWS-5	5.95	0.41	0.01	1.27	5.89	5.63	6.43

Abbreviations: CA – Charlotte's Angel; GWS – Great White Shark; NYC Diesel – New York City Diesel; CBDA – cannabidiolic acid; CBD – cannabidiol; CBN – cannabinol; Δ9-THC - Δ9-tetrahydrocannabinol; Δ9-THCA - Δ9-tetrahydrocannabinolic acid; AVE n=3 – average value from three replicates; BLQ – below limit of quantification, ND – not detected; m/m % - percentage by mass (mass/mass).

Table S7. Reported total CBD content in inflorescences of selected European hemp cultivars and CsW.

Variety / Group	Total CBD (% m/m)	Total Δ9-THC (% m/m)	Reference / Year
W1	5.15 ± 1.41	0.14 ± 0.05	This study
W2	5.14 ± 0.64	0.14 ± 0.02	This study
W3	5.88 ± 0.39	0.16 ± 0.02	This study
W4	5.96 ± 1.18	0.17 ± 0.03	This study
W6	7.61	0.20	This study
Antal	5.70	0.25	[27]; 2021
Białobrzeskie	1.04	NR	[33]; 2021
Carmagnola	4.87	0.24	[27]; 2021
Eletta Campana	4.50	NR	[27]; 2021
Fedora 17	2.60	NR	[28]; 2020
	NR	<0.20	[29]; 2020
	NR	<0.20	[11]; 2026
Felina 32	3.00	NR	[28]; 2020
	NR	<0.20	[29]; 2020
Ferimon	2.85	NR	[28]; 2020
	0.70	0.041	[29]; 2020
Fibranova	4.35	0.26	[27]; 2021
FINOLA	2.32	0.064	[30]; 2019
	NR	NR	[28]; 2020
	NR	<0.20	[12]; 2020
Futura 75	2.48	0.053	[30]; 2019
	2.48	0.06	[31]; 2022
	NR	NR	[28]; 2020
	3.90	<0.20	[11] 2026
Helena	2.40	<0.20	[12]; 2020
KC Dóra	3.63	0.10	[31]; 2022
	3.80	0.22	[11]; 2026
	NR	variable	[12]; 2020
Kompolti	6.45	0.30	[27]; 2021
	7.25	~0.20	[32]; 2025
Kompolti hybrid TC	2.50	<0.20	[12]; 2020
Marina	1.50	<0.20	[12]; 2020
Monoica	NR	<0.20	[12]; 2020
	3.50	0.33	[11]; 2026
Santhica 27	NR	<0.20	[12]; 2020
	~0.20	BLQ	[11]; 2026
Tiborszállási	6.18	0.59	[27]; 2021
	1.60	<0.20	[12]; 2020

	4.30	0.51	[11]; 2026
Tisza	5.84	0.25	[27]; 2021
	1.90	<0.20	[12]; 2020
Tygra	2.58	0.15	[31]; 2022
USO 31	NR	<0.20	[12]; 2020
	NR	<0.20	[29]; 2020
	~1.10	BLQ	[11]; 2026

Abbreviations: W1- W6 - cultivated Macedonian *Cannabis sativa* plant groups from wild-origin (CsW); CBD – cannabidiol; Δ9-THC - Δ9-tetrahydrocannabinol; BLQ – below limit of quantification, ND – not detected, NR - exact numerical value not reported/available in the source data used for this table; m/m % - percentage by mass (mass/mass).

Note: Total CBD = CBD + 0.877 × CBDA and total THC = Δ9-THC + 0.877 × THCA where neutral and acidic forms were reported separately. A value such as <0.20 indicates that the publication explicitly reported the result below that threshold. Approximate (~) values were read from published graphical data and should be replaced by exact numerical values if raw/tabulated data become available.

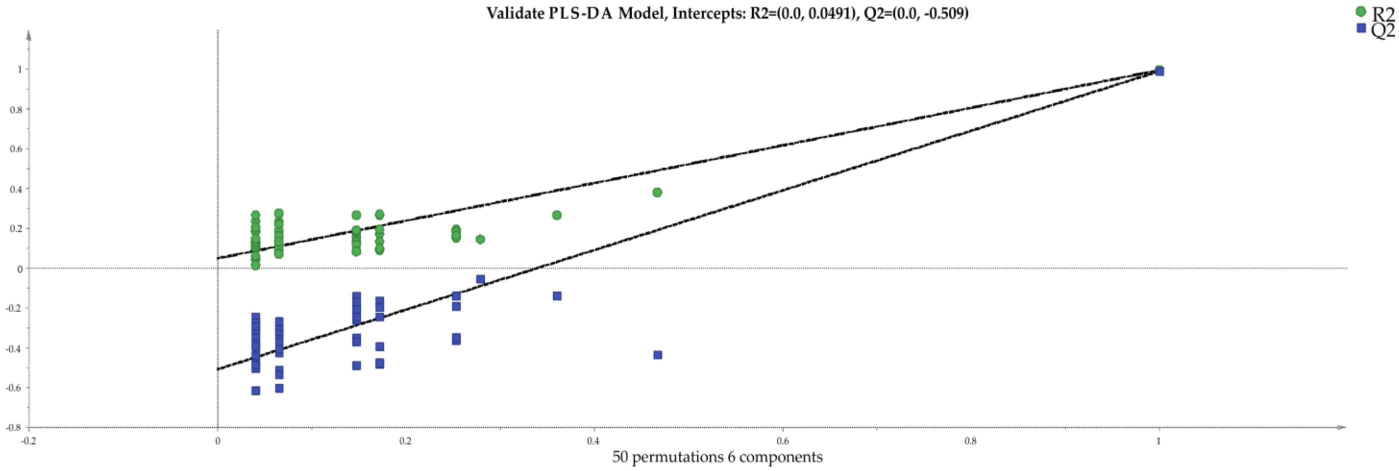

Figure S1. Permutation test ($n = 50$) for the morphology-based PLS-DA model. The model comprised six components and yielded R^2 and Q^2 intercepts of 0.0491 and −0.509, respectively.

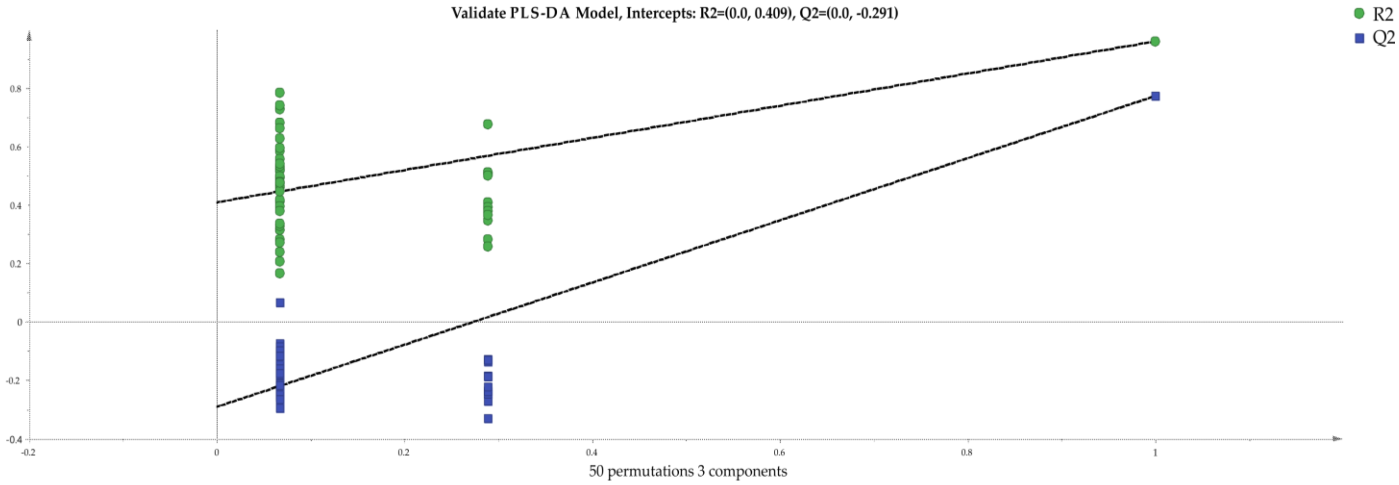

Figure S2. Permutation test ($n = 50$) for the expanded UPOV morphology -based PLS-DA model. The model comprised three components and yielded R^2 and Q^2 intercepts of 0.409 and −0.291, respectively.

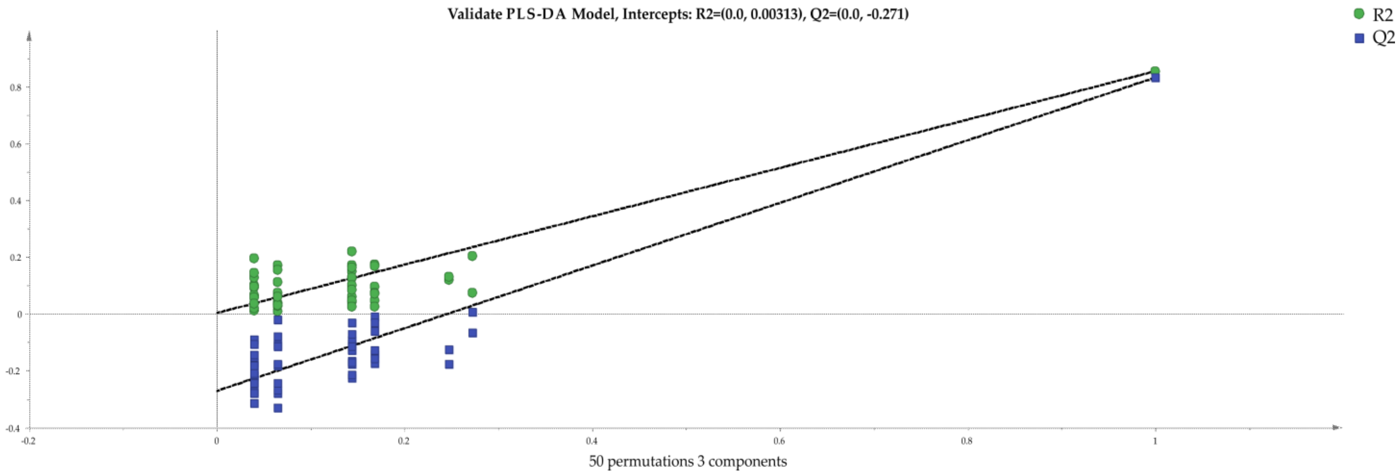

Figure S3. Permutation test ($n = 50$) for the cannabinoid composition based PLS-DA model. The model comprised three components and yielded R^2 and Q^2 intercepts of 0.003 and −0.271, respectively.

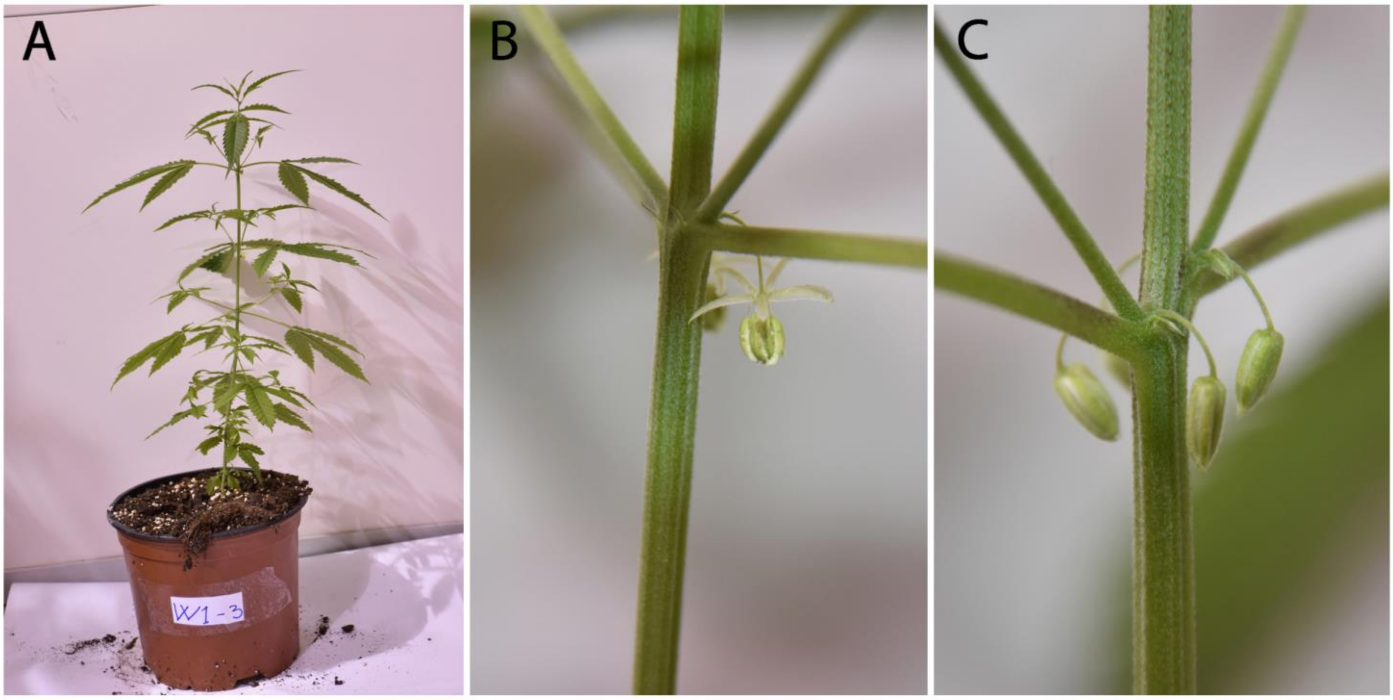

Figure S4. Identification of male plants: (A–C) characteristic morphological features of male plants—racemose inflorescences (inflorescentia racemosa) consisting of multiple small flowers arranged in clusters, with stamens that produce pollen.

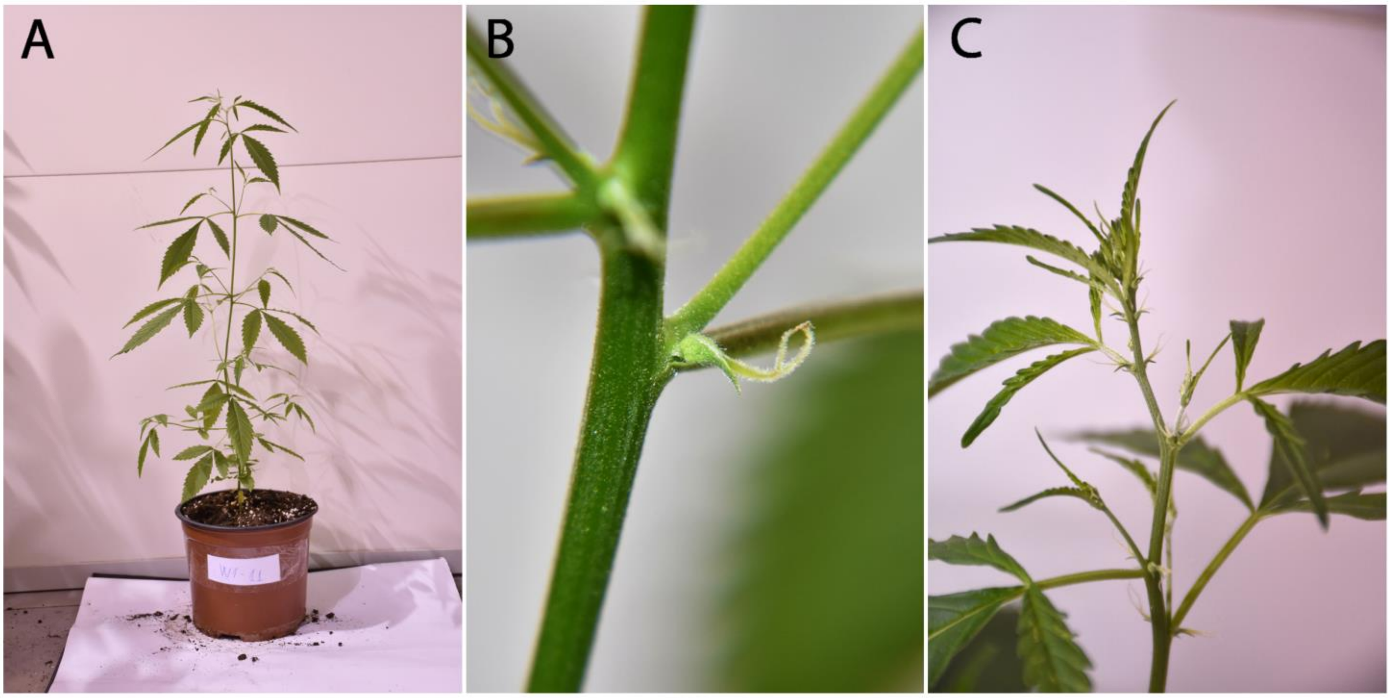

Figure S5. Identification of female plants: (A–C) Characteristic morphological features of female plants—initial flowers developing in the leaf axils (axillae), with pistils bearing characteristically long, hair-like stigmas that capture pollen for fertilization.

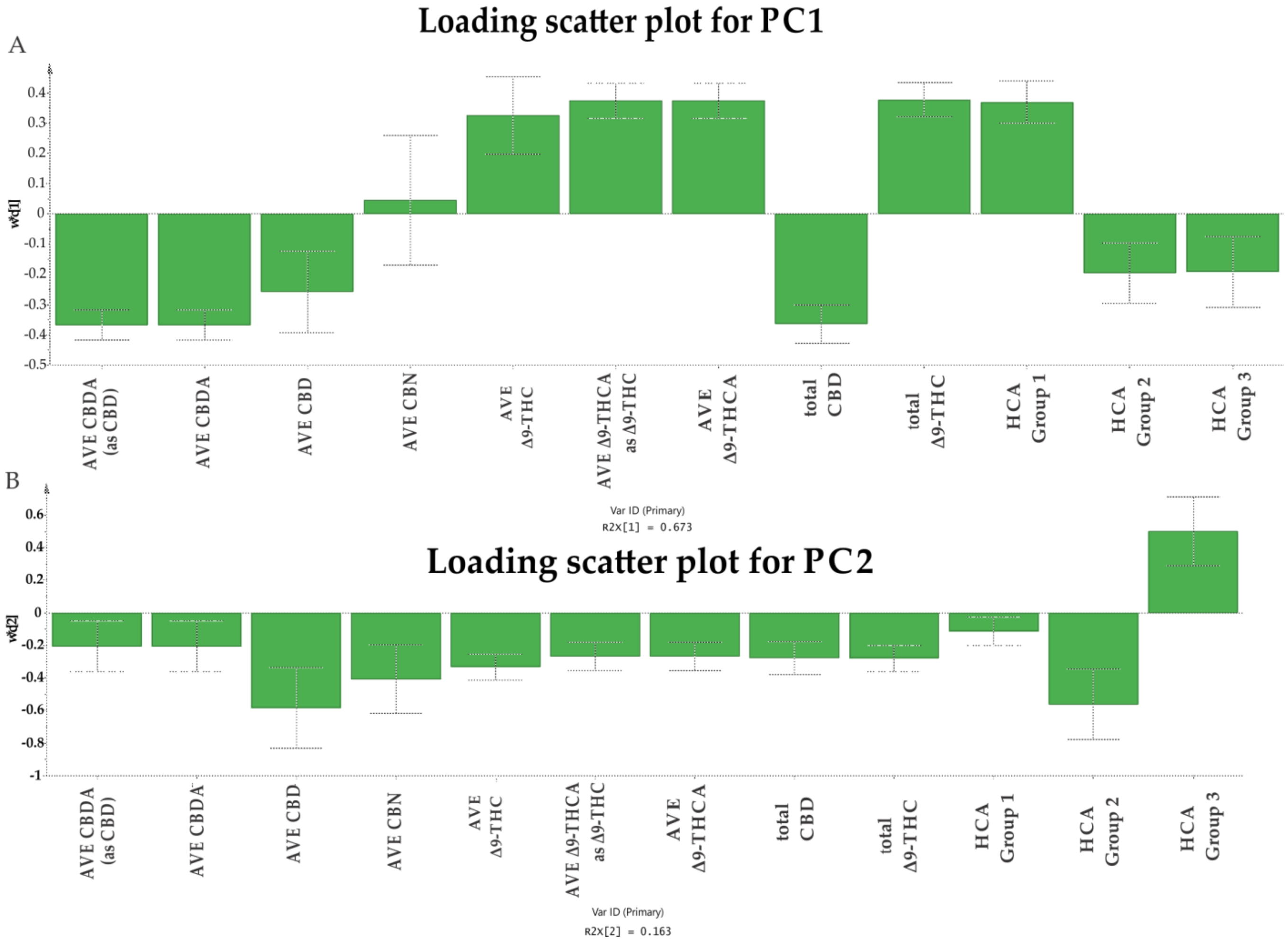

Figure S6. PLS-DA loading plots based on the cannabinoid profiles of cultivated Macedonian *Cannabis sativa* plants from wild-origin (CsW) and commercial cannabis strains, showing the contribution of individual cannabinoids to the observed sample differentiation. Abbreviations: PC – principal component; PLS-DA – partial least square discriminatory analysis; CBDA – cannabidiolic acid; CBD – cannabidiol; CBN – cannabinol; Δ9-THC - Δ9-tetrahydrocannabinol; Δ9-THCA - Δ9-tetrahydrocannabinolic acid; AVE– average.

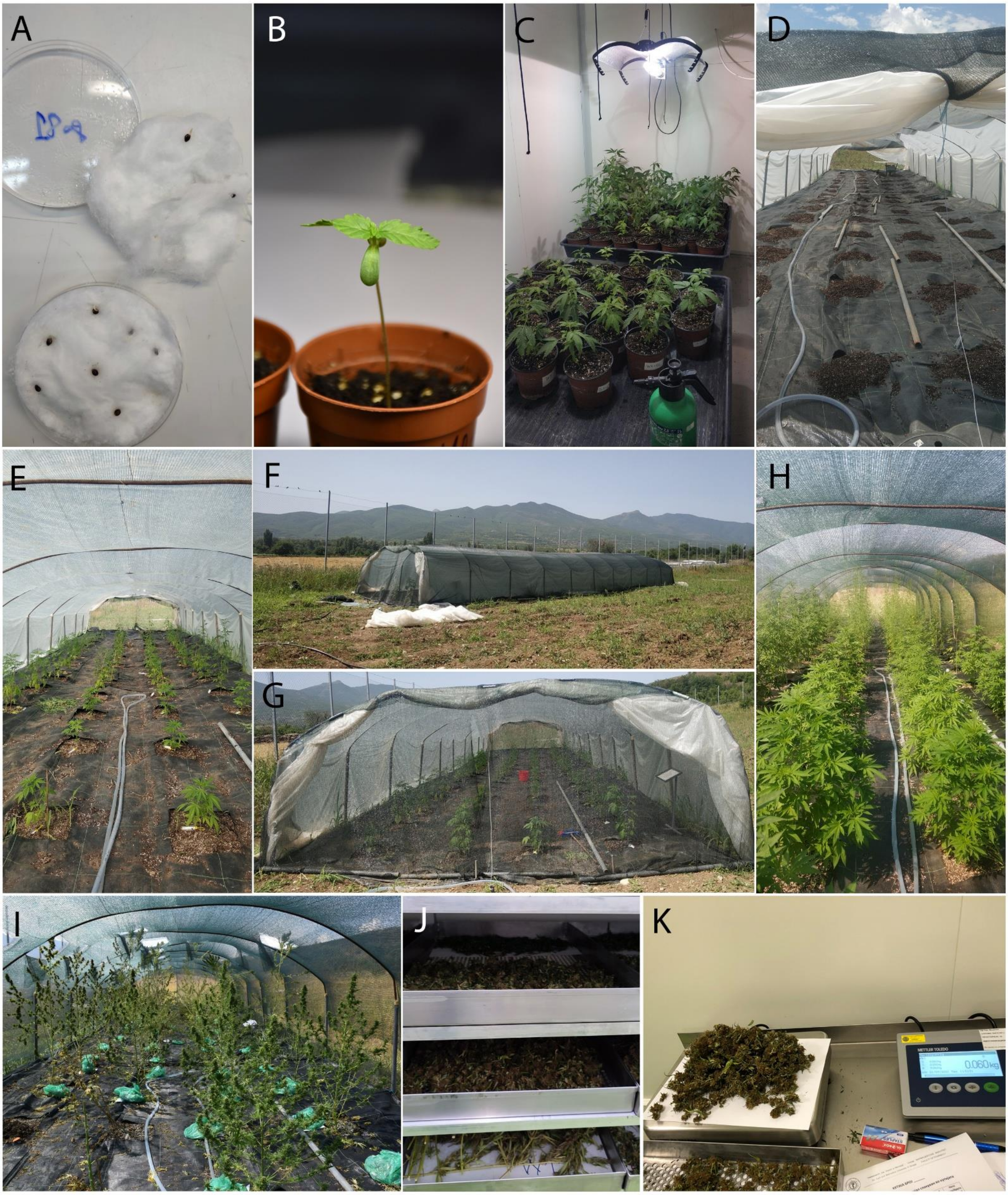

Figure S7. Cultivation process: (a) germination, (b) imbibition, (c) early vegetative stage, (d) preparation of the experimental field for outdoor transplantation, (e) transplanted plants at the experimental field during the early vegetative stage, (f,g) spatial arrangement of the outdoor experimental field, (h) onset of flowering, (i) harvesting, (j) drying, and (k) weighing and packaging of the dried plant material.

References:

11. Flajšman, M.; Kušar, A.; Abramovič, H.; Jakopič, J.; Kocjan Ačko, D.; Čeh, B. Impact of Hemp (Cannabis sativa L.) Variety on the Seed and Stem Yield, Biochemical Characteristics of the Inflorescences and Nutritional Quality of Seeds. Plant Soil Environ. 2026, 72, 122–137. https://doi.org/10.17221/556/2025-PSE.

12. Glivar, T.; Eržen, J.; Kreft, S.; Zagožen, M.; Čerenak, A.; Čeh, B.; Tavčar Benković, E. Cannabinoid Content in Industrial Hemp (Cannabis sativa L.) Varieties Grown in Slovenia. Ind. Crops Prod. 2020, 145, 112082. https://doi.org/10.1016/j.indcrop.2019.112082.

27. Spano, M.; Di Matteo, G.; Ingallina, C.; Botta, B.; Quaglio, D.; Ghirga, F.; Balducci, S.; Cammarone, S.; Campiglia, E.; Giusti, A.M.; et al. A Multimethodological Characterization of Cannabis sativa L. Inflorescences from Seven Dioecious Cultivars Grown in Italy: The Effect of Different Harvesting Stages. Molecules 2021, 26, 2912. https://doi.org/10.3390/molecules26102912.

28. Burgel, L.; Hartung, J.; Pflugfelder, A.; Graeff-Hönninger, S. Impact of Growth Stage and Biomass Fractions on Cannabinoid Content and Yield of Different Hemp (Cannabis sativa L.) Genotypes. Agronomy 2020, 10, 372. https://doi.org/10.3390/agronomy10030372.

29. Ingallina, C.; Sobolev, A.P.; Circi, S.; Spano, M.; Fraschetti, C.; Filippi, A.; Di Sotto, A.; Di Giacomo, S.; Mazzoccanti, G.; Gasparrini, F.; et al. Cannabis sativa L. Inflorescences from Monoecious Cultivars Grown in Central Italy: An Untargeted Chemical Characterization from Early Flowering to Ripening. Molecules 2020, 25, 1908. https://doi.org/10.3390/molecules25081908.

30. Pavlovic, R.; Panseri, S.; Giupponi, L.; Leoni, V.; Citti, C.; Cattaneo, C.; Cavaletto, M.; Giorgi, A. Phytochemical and Ecological Analysis of Two Varieties of Hemp (Cannabis sativa L.) Grown in a Mountain Environment of Italian Alps. Front. Plant Sci. 2019, 10, 1265. https://doi.org/10.3389/fpls.2019.01265.

31. Szalata, M.; Dreger, M.; Zielińska, A.; Banach, J.; Szalata, M.; Wielgus, K. Simple Extraction of Cannabinoids from Female Inflorescences of Hemp (Cannabis sativa L.). Molecules 2022, 27, 5868. https://doi.org/10.3390/molecules27185868.

32. Ioannidis, V.A.; Sygouni, V.; Giannopoulos, S.; Sotirianos, K.; Ioannides, T.; Paraskeva, C.A.; Lamari, F.N. Development of a Validated LC-MS Method for the Determination of Cannabinoids and Evaluation of Supercritical CO2 vs. Ultrasound-Assisted Extraction in Cannabis sativa L. (Kompolti Cv.). Antioxidants 2025, 14, 777. https://doi.org/10.3390/antiox14070777.

33. Pexová Kalinová, J.; Vrchotová, N.; Tříska, J.; Hellerová, Š. Industrial Hemp (Cannabis sativa L.) as a Possible Source of Cannabidiol. J. Cent. Eur. Agric. 2021, 22, 110–118. https://doi.org/10.5513/JCEA01/22.1.2860.
